# FunVIP: Fungal Validation and Identification Pipeline based on phylogenetic analysis

**DOI:** 10.71150/jm.2411017

**Published:** 2025-04-29

**Authors:** Chang Wan Seo, Shinnam Yoo, Yoonhee Cho, Ji Seon Kim, Martin Steinegger, Young Woon Lim

**Affiliations:** 1 School of Biological Sciences, Seoul National University, Seoul 08826, Republic of Korea; 2 Institute of Biodiversity, Seoul National University, Seoul 08826, Republic of Korea; 3 Institute of Molecular Biology and Genetics, Seoul National University, Seoul 08826, Republic of Korea; 4 Artificial Intelligence Institute, Seoul National University, Seoul 08826, Republic of Korea

**Keywords:** automatic pipeline, DNA barcoding, fungal identification, mislabeled sequences, sequence validation, tree interpretation

## Abstract

The increase of sequence data in public nucleotide databases has made DNA sequence-based identification an indispensable tool for fungal identification. However, the large proportion of mislabeled sequence data in public databases leads to frequent misidentifications. Inaccurate identification is causing severe problems, especially for industrial and clinical fungi, and edible mushrooms. Existing species identification pipelines require separate validation of a dataset obtained from public databases containing mislabeled taxonomic identifications. To address this issue, we developed FunVIP, a fully automated phylogeny-based fungal validation and identification pipeline (https://github.com/Changwanseo/FunVIP). FunVIP employs phylogeny-based identification with validation, where the result is achievable only with a query, database, and a single command. FunVIP command comprises nine steps within a workflow: input management, sequence-set organization, alignment, trimming, concatenation, model selection, tree inference, tree interpretation, and report generation. Users may acquire identification results, phylogenetic tree evidence, and reports of conflicts and issues detected in multiple checkpoints during the analysis. The conflicting sample validation performance of FunVIP was demonstrated by re-iterating the manual revision of a fungal genus with a database with mislabeled sequences, *Fuscoporia*. We also compared the identification performance of FunVIP with BLAST and q2-feature-classifier with two mass double-revised fungal datasets, *Sanghuangporus* and *Aspergillus* section *Terrei*. Therefore, with its automatic validation ability and high identification performance, FunVIP proves to be a highly promising tool for achieving easy and accurate fungal identification.

## Introduction

The kingdom *Fungi* encompasses an estimated 2.2–3.8 million (Hawksworth and Lücking, 2017) or up to 12 million species (Bhunjun et al., 2022), of which 161,455 species names have been currently accepted (www.speciesfungorum.org/Names/Names.asp, accessed in 2024. 10. 07). Fungi exhibit various trophic modes (such as saprotrophs, endophytes, and pathogens) and growth forms (including mycelia and yeasts) across diverse environments (Alexopoulos et al., 1996; Nguyen et al., 2016; Tedersoo et al., 2014). Fungi have evolved to adapt to diverse environments by producing various enzymes and metabolites. These characteristics have facilitated the study of fungi in various fields of research, such as agriculture, ecology, pathology, and pharmacology (Köhler et al., 2017; Nilsson et al., 2019a). Misidentification of fungal species is causing inappropriate application in the industry (Lücking et al., 2021; Tomé et al., 2019) and incorrect diagnosis and treatment in health care (Spivak and Hanson, 2018) and forms a threat to food safety and public health (Frisvad et al., 2006; Wei et al., 2022). Thus, accurate species identification is required for scientific communication in and across these fields. Traditional fungal identification relied on morphological and phenotypic characteristics (Hawksworth et al., 1996; Kandawatte et al., 2021), but complex growth morphologies, sexual dimorphism, convergent evolution, and phenotypic plasticity have made precise fungal identification difficult (Atkins and Clark, 2004; Hibbett and Taylor, 2013; Taylor, 2011).

To address these challenges, the reliance on molecular identification methods has increased in fungal systematics since the early 1990s (Hebert et al., 2003; Hibbett, 1992; White et al., 1990). Compared to morphology, DNA sequences offer more independent characters, leading to a more robust estimates of fungal phylogeny (Hillis, 1987). In 2012, the internal transcribed spacer (ITS) was designated as a universal barcode marker for the kingdom *Fungi* (Schoch et al., 2012). Since then, a standardized sequencing protocol for the barcode sequence has led to a substantial accumulation of DNA sequence data in global public databases, such as GenBank (Sayers et al., 2023) and UNITE (Nilsson et al., 2019b). The increase of sequence data in public nucleotide databases has made DNA sequence-based identification an indispensable tool for fungal species identification (Chase and Fay, 2009; Hibbett et al., 2016). The Basic Local Alignment Search Tool (BLAST) (Camacho et al., 2009) is a widely utilized method for sequence-based identification, recognized for its standardization and accessibility (Kõljalg et al., 2005; Sokoł et al., 1999). Using an accurate database is crucial for achieving reliable BLAST results (Hawksworth, 2004; Meiklejohn et al., 2019; Somervuo et al., 2016). However, a large proportion of data in public databases is inaccurate (up to 30%) (Hofstetter et al., 2019; Lücking et al., 2020a; Nilsson et al., 2006) causing widespread and frequent misidentifications in fungal identifications (Tedersoo et al., 2022).

Phylogeny-based identification offers a solution to prevent misidentifications caused by databases with mislabeled sequences. Even so, issues like inappropriate genetic marker selection can mislead the user (Lücking and Hawksworth, 2018). Extensive taxonomic knowledge and the ability to evaluate a phylogenetic tree are required to recognize faulty data and accurately identify a species. Moreover, the lack of standardization in phylogeny-based identification may result in different outcomes depending on the user, even with the same input file. This reduces the productivity, scalability, and reproducibility of phylogeny-based identification (Raja et al., 2017). Therefore, to achieve a uniform and accurate result of phylogeny-based identification, objective decisions should be made in steps such as database collection, outgroup selection, trimming, alignment editing, and phylogenetic tree interpretation. Developing a standardized phylogeny-based identification method would improve accuracy and reduce reliance on expertise.

Efforts to develop an automated phylogenetic pipeline aimed to standardize the species identification processes. The automated phylogenetic pipeline applicable for species identification began with Phylogeny.fr (Dereeper et al., 2008) and recently advanced to One-click Fungal Phylogenetic Tool (OFPT) (Zeng et al., 2023). However, these tools require users to interpret the phylogenetic tree outcome for species identification, thus not being fully automated. Fully automated phylogenetic pipelines, such as SUPERSMART (Antonelli et al., 2017) and OneTwoTree (Drori et al., 2018) were designed to examine the evolutionary relationship among well-established species. However, these tools are impractical for identification purposes. Regarding mislabeled data, the Semi-Automatic Taxonomic Improvement and Validation Algorithm (SATIVA) (Kozlov et al., 2016) validates conflicting phylogenetic trees but has not been integrated into an automatic identification pipeline. Thus, at present, there is no rapid, practical tool available for identifying fungal species in a phylogenetic context (Table 1).

To address the current challenges in fungal identification, we developed FunVIP, an automated phylogeny-based validation and identification pipeline for fungi. This pipeline only requires one table-styled query file from the user, one table-style file of database references, and a single command line. The user can retrieve data at any checkpoint (e.g., alignment, trimming, and the raw tree inference step) and inspect the result. In this paper, we demonstrate the validation ability and identification accuracy of FunVIP through three dataset examples: *Fuscoporia* (*Basidiomycota*) dataset (Cho et al., 2023), *Sanghuangporus* (*Basidiomycota*) dataset (Shen et al., 2021), and *Aspergillus* section *Terrei* (*Ascomycota*) dataset (Wu et al., 2022). These datasets contain numerous misidentifications but have recently undergone manual re-identification and revision based on phylogenetic analysis by taxonomic experts. We present the validation performance of FunVIP through analysis of the original *Fuscoporia* dataset to evaluate the ability of FunVIP to recognize mislabeled samples. Additionally, we demonstrate the identification performance of FunVIP by analyzing the original *Sanghuangporus* and *Aspergillus* section *Terrei* datasets. We compare the results with BLAST and q2-feature-classifier to highlight the superior identification accuracy of FunVIP. According to our findings, FunVIP facilitates user-friendly fungal identification based on phylogenetic analysis while minimizing errors.

## Materials and Methods

### Workflow

**Fig. 1.**
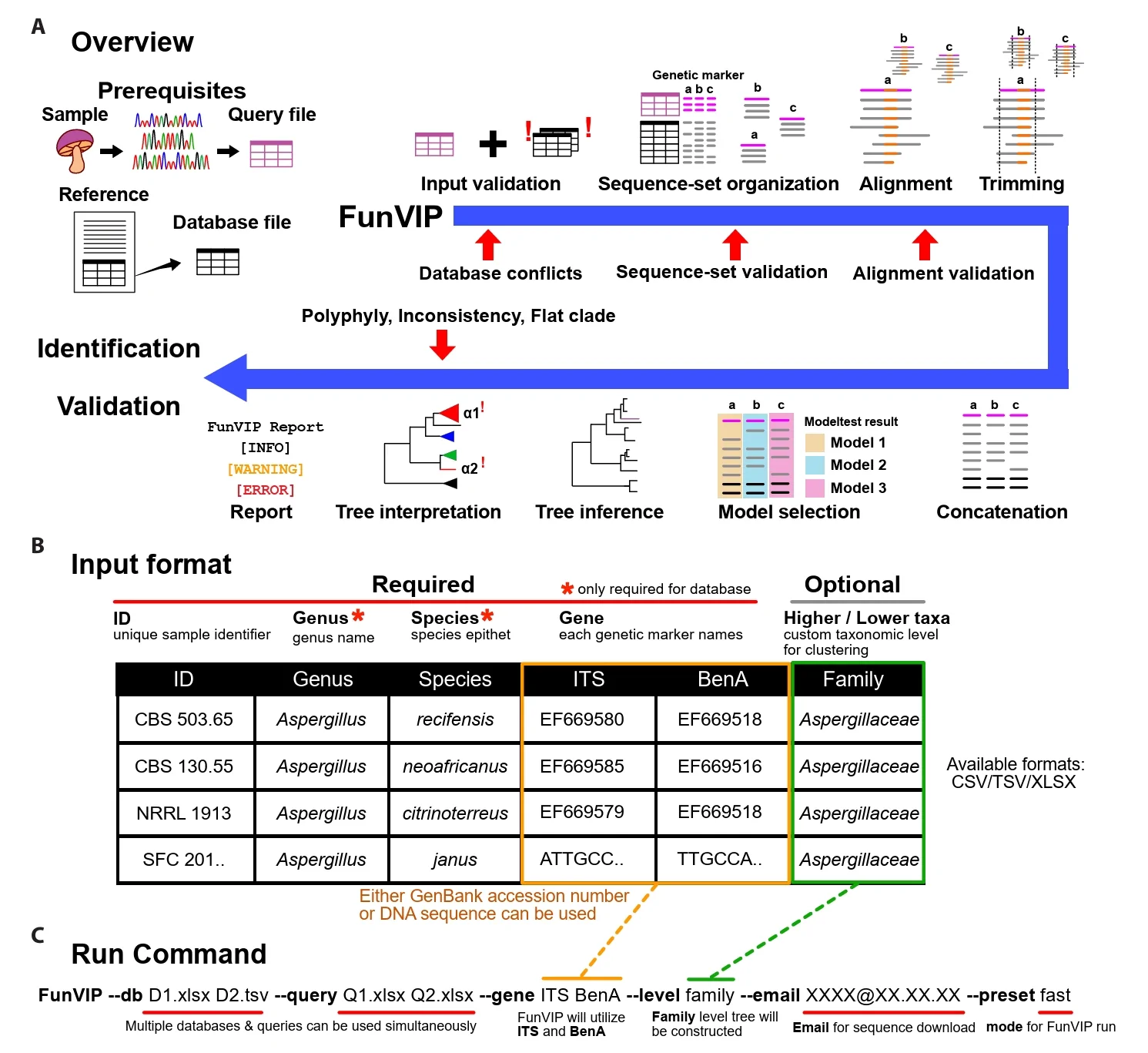
FunVIP workflow and usage. (A) Schematic representation of the phylogenetic tree-based identification using FunVIP. The red arrows indicate validation checkpoints in each process, and the blue arrow indicates the FunVIP pipeline. Small letters denote each genetic marker. (B) Database and query input format example with descriptions for FunVIP. Columns marked with red asterisks are mandatory to fill for the database file, and optional for the query file. (C) Example command and its description to execute FunVIP.

**Implementation of FunVIP:** FunVIP is an automated pipeline for fungal identification and validation based on phylogenetic analysis. Within a single execution of FunVIP, nine steps are included: input validation, dataset generation, alignment, trimming, concatenation, model selection, tree inference, tree interpretation, and report generation (Fig. 1A). Single node hardware with at least 8 GB is required to run FunVIP, depending on the size of the dataset and the number of threads to be utilized. The pipeline is mostly written in Python 3, and it is compatible with Windows, Mac (Apple Silicon), and Linux platforms. A Conda environment is required to install and execute FunVIP.

**Prerequisites:** FunVIP requires a table of query files, a table of database files, and a single command line string as input (Fig. 1A). The query table contains unique sample IDs (identifiers indicating biological individual, e.g., strain number or specimen number) and sequences for each genetic marker (GenBank accession numbers or nucleotide sequences). The genus name and species epithet columns can optionally be applied to the query for additional comparison of FunVIP-based identification with the given taxonomy annotation. The database table contains sample IDs, sequences of each gene name, genus name, species epithet, and optional metadata (Fig. 1B). The run command consists of the designation of query and database table files and applicable options, such as mode (fast or accurate) (Fig. 1C). For advanced usage, users can select options for each step from a list of possible choices, with the first option set as the default option (Supple Data 1). Detailed instructions and tips for preparing database and query files for FunVIP is available on tutorial page (https://github.com/Changwanseo/FunVIP/blob/main/tutorial/tutorial2.md).

**Input validation step:** FunVIP performs input validation to detect and curate human errors before taxonomic analysis. Specifically, FunVIP detects and curates invalid characters in IDs, conflicting sample information, invalid sequence format, and mislabeled genetic markers for both database and query files. For the ID curation, FunVIP automatically corrects characters causing errors during pipeline execution (e.g., umlauts, Latin small ligature “fi”, non-breaking spaces, and whitespaces). In cases of conflicting sample information, where identical sample IDs are associated with different sequences or genetic markers, FunVIP reports the conflicts to guide users in resolving them. For sequence format validation, FunVIP determines whether the sequence is provided as a GenBank accession, DNA string, or FASTA format (Pearson and Lipman, 1988). If a GenBank accession is provided, FunVIP checks whether the accession is valid and retrieves the corresponding sequence using GenMine (Seo et al., 2022). When sequences are provided as raw DNA strings, FunVIP checks for invalid DNA bases. When genetic marker is not given, FunVIP uses BLAST or MMseqs2 (Steinegger and Söding, 2017) to assign the appropriate genetic marker, and excludes invalid data from further analysis.

**Sequence-set organization step:** Many fungal barcodes cannot be reliably aligned over certain taxonomic levels (Anslan et al., 2018; Tedersoo et al., 2022), dataset division by taxonomic level is required for accurate phylogenetic analysis (Supple Data 2.1, Fig. S2.1). Also, topological incongruences across genetic marker trees should be compared to find out potential mislabels and data contamination in genetic marker annotation. In the sequence-set organization step, FunVIP reorganizes given database and query sequences by taxonomic group-genetic marker combinations to address these needs (Supple Data 2.1, Fig. S2.1).

FunVIP performs sequence-set organization with the consideration that the given database may include mislabeled sequences. For each sequence-set, ingroup, suspicious and outgroup sequences are collected from databases (Supple Data 2.3, Fig. S2.3). Suspicious sequences refer to potential ingroup sequences that are suspected to be misannotated as other taxa (Supple Data 2.4, Fig. S2.4). Outgroup sequences, validated to be outside the target taxonomic group, are sorted into the sequence-set (Supple Data 2.5, Fig. S2.5). Query sequences are sorted into the appropriate sequence-set based on their closest taxonomic assignment. The performance of the query sequence assignment was validated with UFCG dataset (Supple Data 2.6, Fig. S2.6). All sequence distance calculations required for assigning sequences to sequence-sets are determined by pairwise BLAST or MMseqs match. Checkpoints throughout the sequence-set organization step report warnings for users if any issue arises or if the database is insufficient.

**Multiple sequence alignment, trimming, concatenation, model selection, and tree inference steps:** FunVIP aligns each sequence-set using MAFFT version 7 (Katoh and Standley, 2013) with --maxiterate 1000 option for iterative refinement. Reverse complementary sequences are automatically redirected using --adjustdirection option of MAFFT. The alignments are trimmed using trimAl (Capella-Gutiérrez et al., 2009) or Gblocks (Castresana, 2000). To maximize the number of informative characters, complementary scripts that prevent trimming non-terminal columns of the alignments are included (Supple Data 3.1, Fig. S3.1). A checkpoint in the trimming step assesses whether the alignment meets the criteria necessary infer a valid phylogenetic tree, requiring at least four sequences and at least one fully aligned column across all the alignments (Supple Data 3.2, Fig. S3.2). The Transitive Consistency Score (TCS) (Chang et al., 2014) is calculated with T-Coffee (Notredame et al., 2000) to filter out misaligned sequences. Concatenated multiple genetic marker matrices are generated for each taxonomic group. The nucleotide substitution model for partitions is selected and applied through ModelTest-NG (Darriba et al., 2020) or ModelFinder (Kalyaanamoorthy et al., 2017). Phylogenetic trees are inferred from both individual genetic markers and concatenated matrices using FastTree 2 (Price et al., 2010), IQ-TREE 2 (Minh et al., 2020), or RAxML version 8 (Stamatakis, 2014).

**Fig. 2.**
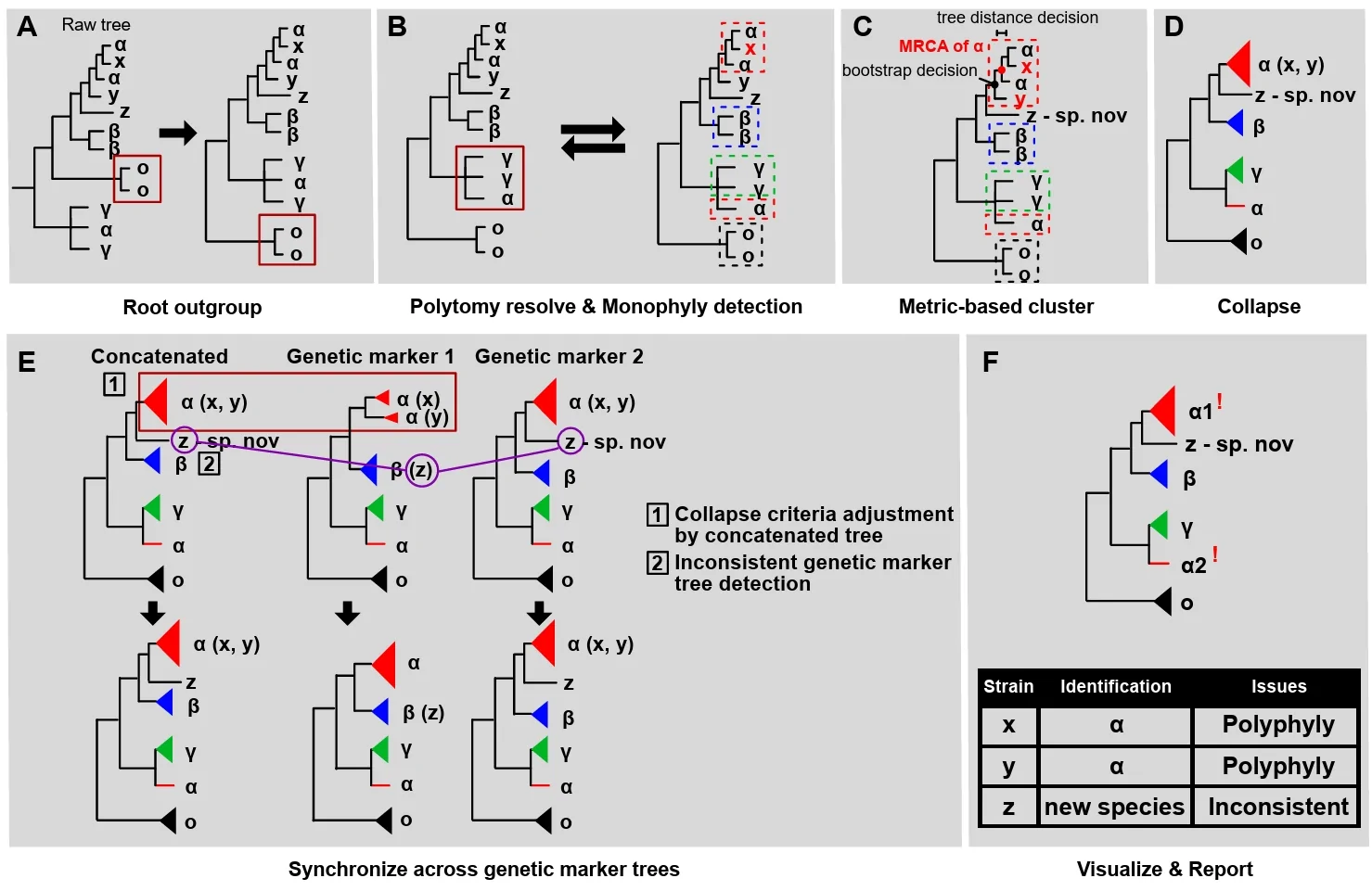
Tree interpretation algorithms performed in FunVIP. Infographics describing algorithms performed during the tree interpretation step. Taxa α, β, and γ indicate database samples, O indicates database samples selected as the outgroup, and x, y, and z indicate query samples. Red, blue, and green colors indicate taxa α, β, and γ respectively. (A) Rooting of outgroup. Crimson squares indicate the outgroup. (B) Polytomy-resolving and monophyly detection. The crimson square indicates polytomy of taxon γ, and dotted squares indicate species’ borders defined by monophyly detection. The bidirectional arrow indicates recursive calls between the two functions. (C) Metric (tree distance, bootstrap)-based clustering. Dotted squares indicate species’ borders adjusted by metric-based clustering. MRCA stands for the Most Recent Common Ancestor. (D) Collapsing. (E) Synchronizing across each genetic marker tree. The crimson square indicates different criteria applied for collapsing by each genetic marker tree. Purple circles indicate sample z assigned to different species by each genetic marker tree. (F) Visualizing and reporting.

**Tree interpretation step:** The tree interpretation step involves rooting of the outgroups, polytomy (non-bifurcating branches) resolving, monophyly detection, metric-based clustering, collapsing, synchronizing, visualizing, and reporting (Fig. 2, Supple Data 4, Fig. S4.1). The tree interpretation step deals with three kinds of potential issues: flat branches (Fig. 2B), polyphylies, and inconsistencies (Fig. 2F). Flat branches (polytomies with zero-length branches) are inspected to determine whether the genetic marker has sufficient resolution to resolve taxa at the species level. Polyphylies (multiple monophyletic clades annotated under an identical taxon name) are numbered to inform users to check for misidentified species in the database. Inconsistencies (identification obtained from each of the genetic marker trees differing from the concatenated tree) are inspected to check for mislabeled genes. FunVIP generates a report in table format for the identification result and all potential issues detected during the pipeline run, allowing users to recognize and take appropriate action. It also visualizes phylogenetic trees in a vector image format suitable for further editing.

### Performance evaluation

All processes in performance evaluation section were performed with Ryzen 3900X (12 cores, 24 threads) processor and 64 GB memory with Ubuntu 20.04 environment.

**Database validation performance of FunVIP:** Validation ability is defined by finding misannotated sequences within the given dataset. To demonstrate the database validation ability of FunVIP, the *Fuscoporia* dataset was selected as a case study, as it has numerous incorrect identifications, making it an ideal example to illustrate these issues. Taxonomic data tables from seven references indicated in Table 1 of Cho et al. (2023) were prepared. All tables were reformatted to be suitable for valid FunVIP input. The database tables from the seven references were merged into one file for better manipulation. Detailed processes held to prepare input for the analyses are described in Supple Data 5.1.

**Fig. 3.**
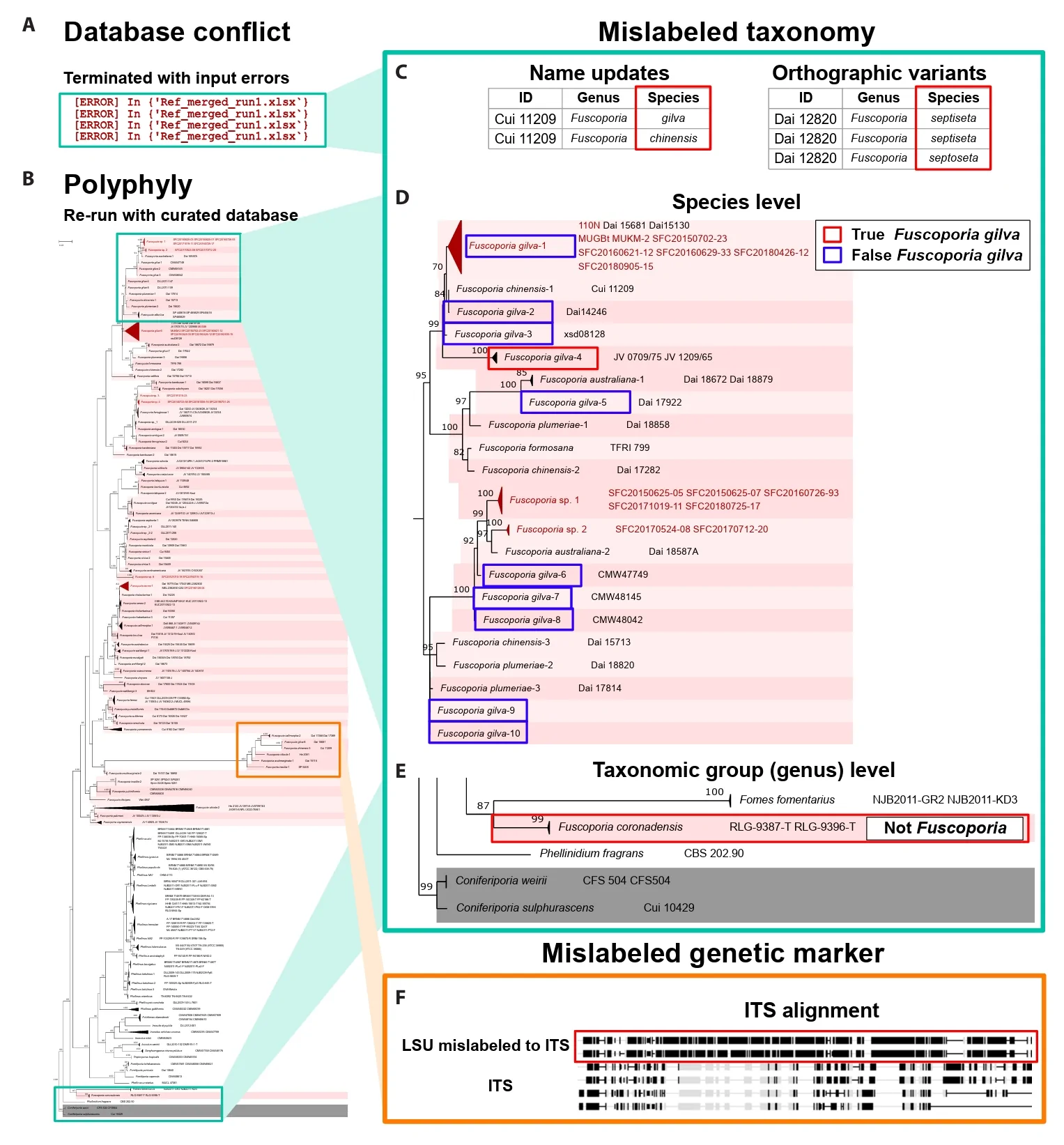
Validation ability of FunVIP on re-analyzing the *Fuscoporia* dataset. Mint-colored squares indicate mislabeled taxonomy and orange-colored squares indicate mislabeled genetic markers. (A) The initial FunVIP run failed due to database conflicts. (B) Phylogenetic tree generated with polyphyly issues detected by FunVIP using the curated *Fuscoporia* database. (C) Mislabeled taxonomy detected from database conflicts. Red squares indicate conflicting species epithets among the samples in the database. (D) Mislabeled sample found by polyphyly at the species level. The red square indicates true *F. gilva*, and the blue squares indicate mislabeled *F. gilva*. (E) Mislabeled sample found by polyphyly at the taxonomic group (genus) level. (F) Samples with mislabeled genetic markers (ITS and LSU switched) were found by polyphyly. The black color in the alignment indicates unaligned regions to consensus, and the grey color in the alignment indicates aligned regions to consensus. Red squares indicate LSU sequences mislabeled as ITS.

The first run of FunVIP was performed with the database file and query file in fast mode. Database conflicts (incongruent genus, species, or sequence information among database samples with identical IDs) were found during the input validation step and recorded to the log file (Fig. 3A). The database file was curated according to the conflict report from the first run. For every conflict, the decision of the original reference was applied. The second run was performed with the curated database file and query file in fast mode, and polyphylies found in the concatenated tree were counted from the “ISSUES” column of the report file (Fig. 3B). Detailed processes held during the analyses are described in Supple Data 5.2.

**Identification performance of FunVIP:** To assess the performance of FunVIP in species identification, we compared FunVIP with the results of BLAST searches (Camacho et al., 2009) and the q2-feature-classifier (Bokulich et al., 2018) using two fungal sequence datasets: *Sanghuangporus* in *Basidiomycota* (Shen et al., 2021) and *Aspergillus* section *Terrei* in *Ascomycota* (Wu et al., 2022). These datasets represent sequences of fungal taxa that have been re-identified and revised by taxonomic experts based on phylogenetic analysis. We selected these datasets for evaluating identification performance because expert-revised taxonomic annotation is essential for an accurate identification performance assessment.

The *Sanghuangporus* dataset comprised ITS sequences of 271 samples, including 15 database and 256 query samples collected from the study by Shen et al. (2021). Two *Tropicoporus* sequences were included as outgroup samples (Wu et al., 2022). The *Aspergillus* section *Terrei* dataset comprised data from four genetic markers [ITS, β-tubulin (*BenA*), calmodulin (*CaM*), and RNA polymerase II (*RPB2*)], including 56 database (outgroup included) and 79 query samples collected from the study by Barros et al. (2020). Manual correction of both datasets, such as orthographic variants and mislabels were performed (Supple Data 6.1, 6.2).

To compare identification performance, multiple options were used to evaluate consistency in each analysis (Supple Data 6.3). A combination of --preset (fast or accurate) and --collapsedistcutoff (0.005–0.3) options were used for FunVIP. The local version of BLAST was performed with database and query files containing the same contents to control variables. Species of the BLAST match with the highest percent identity and within the selected percent identity cutoff (99.5–70) were selected as the identification result. The taxonomic assignment results of q2-feature-classifier were directly applied for the identification result. Accuracy, precision, recall, and F1 score were utilized as statistics to evaluate the performance of identification. Due to the limitation of BLAST and q2-feature-classifier, delimitation of individual new species (e.g., sp. 1 and sp. 2) is not accounted for in the statistics (all regarded as “sp.”). The Mann-Whitney U test was performed for each pair of mean and standard deviation of accuracies to assess the statistical significance.

## Results

### Validation performance of FunVIP

A total of 501 database samples and 22 query samples were applied in the first run of FunVIP with the *Fuscoporia* dataset. A total of 113 database conflicts were found during the first run, including ten errors and 103 minor conflicts. The ten errors were caused by six name updates in the subsequent reference (i.e. *F. wahlbergii* re-identified as *F. australasica*) and four orthographic variants (i.e. *F. setifer* as a typo of *F. setifera*) (Fig. 3C). The other 103 minor conflicts found in the database included two cases where partial sequences with identical sample ID but different accessions (i.e. JQ794579 and JQ794580 both uploaded as the ITS sequence of sample JV0610/14PK). The remaining 101 cases involved sequences that were differentially present among the same sample ID [i.e., the *TEF1* sequence is present only for the Cui 11801 sample in the latter reference (Du et al., 2020) and is absent in prior reference (Chen and Dai, 2019)].

The number of samples in the database decreased from 501 to 356 after applying curation according to the conflicts found in the first run and removing duplicates. Among the 356 database samples, 108 samples representing polyphyly were found from the concatenated tree, comprising 20 species and 50 clades. Among the 108 polyphyly samples, 100 samples (44 clades) were caused by mislabeled taxonomies (i.e., *F. gilva* divided into 10 different clades, with only clade four remaining as the actual *F. gilva* species) (Fig. 3D). Two samples from one species (annotated as *F. coronadensis*) turned out to be non-*Fuscoporia* (Fig. 3E). The remaining seven samples (six clades) were revealed to have mislabeled genetic marker issues, where LSU sequences were labeled as ITS sequences (Fig. 3F).

### Identification performance evaluation

**Fig. 4.**
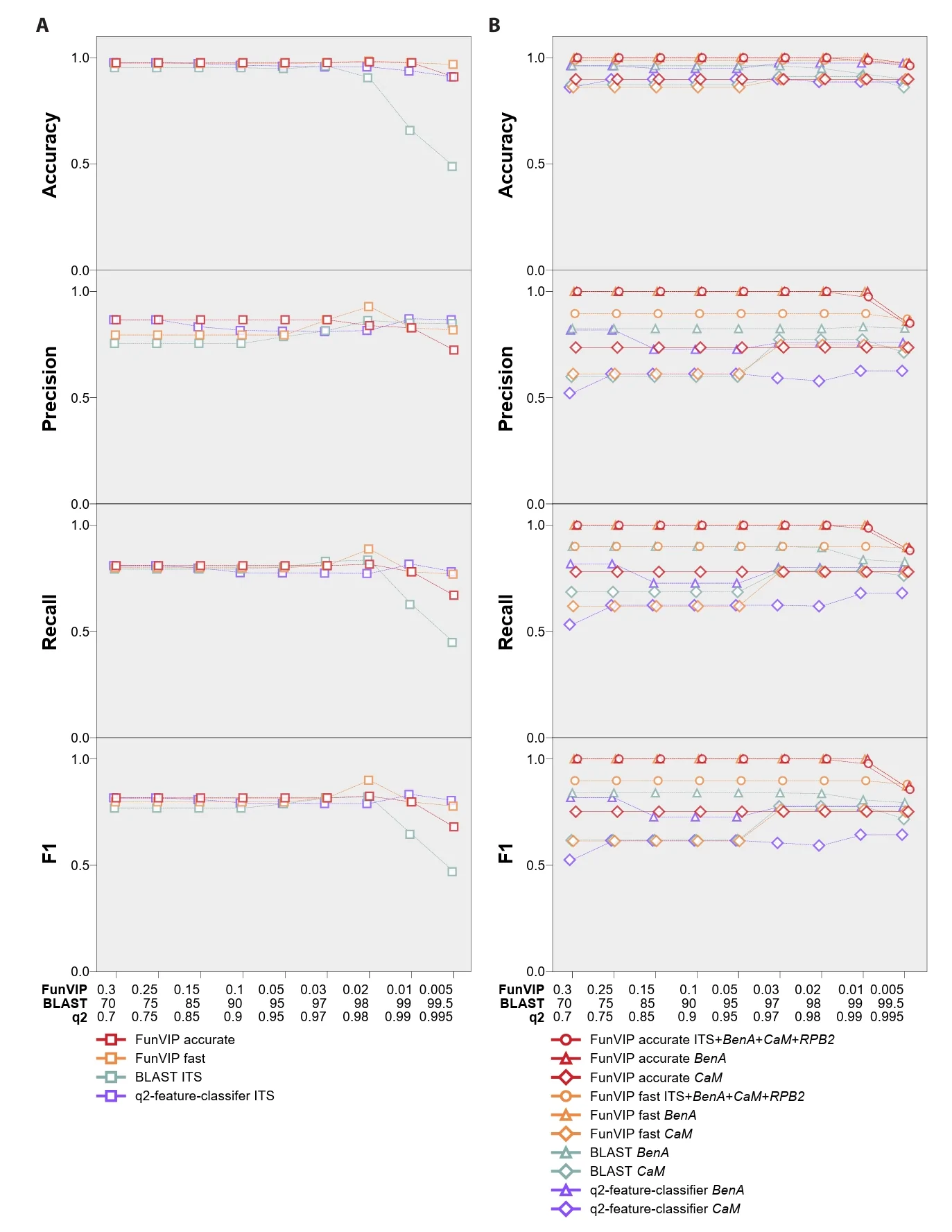
Accuracy, precision, recall, and F1 score for FunVIP, BLAST, and q2-feature-classifier using the *Sanghuangporus* and *Aspergillus* section *Terrei* datasets. (A) Multi-classifier metrics of the *Sanghuangporus* dataset using the ITS region. (B) Multi-classifier metrics of the *Aspergillus* section *Terrei* dataset with the multiple genetic markers, *BenA* and *CaM*. The analyses included FunVIP-fast, FunVIP-accurate, BLAST, and q2-feature-classifier. Individual data points of the analyses are represented by dots. The X-axes indicate options that were applied to each of the analyses (FunVIP – collapsedistcutoff, BLAST – percent identity cutoff, q2-feature-classifier – confidence threshold). The Y-axes indicate each of the metrics in the title of the graphs.

FunVIP exhibited better performance across the majority of the four metrics assessed compared to BLAST and q2-feature-classifier (Fig. 4, Supple Data 7, Table S7.1–7.4). In the *Sanghuangporus* dataset, the mean identification accuracies were 94.25% in the fast mode and 97.28% in the accurate mode, both of which significantly surpassed the 87.24% performance of BLAST and slightly better than the 95.67% performance of q2-feature-classifier (Fig. 4A). When using the default option of FunVIP (--collapsedistcutoff 0.01) in the accurate mode, six out of 254 entries (2.24%) were misidentified due to systematic underclassification [*Sanghuangporus* sp. 3 misidentified as *S. microcystideus* (n=4)] and overclassification [*S. weirianus* identified as *Sanghuangporus* sp. 2 (n=1), *S. zonatus* identified as *Sanghuangporus* sp. 4 (n=1)]. The standard deviation of accuracy was lower for FunVIP (fast: 0.0042, accurate: 0.0212) than for BLAST (0.1552), and similar to those of q2-feature-classifier (0.0203) indicating that FunVIP is more consistent than BLAST.

For the *Aspergillus* section *Terrei* dataset, FunVIP achieved mean identification accuracies of 98.6% in the fast mode and 99.4% in the accurate mode, significantly outperforming BLAST with *BenA* (95.1%) and with *CaM* (88.4%) (Fig. 4B). FunVIP also achieved significantly better performance than q2-feature-classifier with *BenA* (94.9%) and *CaM* (88.5%). Moreover, the standard deviations of accuracy across multiple options (0.0040 in fast mode, 0.0121 in accurate mode) were significantly lower for FunVIP than for BLAST (*BenA*: 0.0215, *CaM*: 0.0193), and similar to those of q2-feature-classifier (0.0111, 0.0119). In the default option of FunVIP (--collapsedistcutoff 0.01) in the accurate mode, only one sample, URM3371 *A. recifensis* was misidentified to *Aspergillus* sp. 1.

## Discussion

The demands for precise fungal identification are increasing across diverse scientific fields, including taxonomy, medical and nutritional sciences, clinical practice, and quarantine management. However, consistent generation of mislabeled sequences poses a significant obstacle to accurate sequence-based identification. To address this challenge, we developed FunVIP to provide a user-friendly and intuitive solution for species identification.

### FunVIP: a superior species identification pipeline based on phylogeny

Practical automated phylogeny-based pipelines fungal identification pipelines are scarce due to challenges in standardization and expertise requirements. A standardized phylogenetic analysis is important for objective identification and cross-validation (Dissanayake et al., 2020). However, variables such as alignment editing, trimming, and branch length criteria for species delimitation are often subjective, which can lead to irreproducible and incoherent phylogenetic trees (Petersen et al., 2010). FunVIP addresses these challenges by allowing users to define ambiguous variables digitally and apply advanced options that can be saved as preset files for reproducibility (Supple Data 1). For instance, alignment editing and trimming can be numerically specified using options such as --mafft-algorithm, --mafft-op, --mafft-ep, --trimal-algorithm, --trimal-gt, and --allow-innertrimming, with outputs available for comparison. Subjective criteria for species delimitation, such as branch length and bootstrap support, can be uniformly defined using the --collapsedistcutoff and --collapsebscutoff options. The --continue option enables users to reproduce phylogenetic analyses and objectively assess the impact of different variables by modifying options in subsequent runs.

Standardization can also contribute to reproducible phylogenetic analysis. Being able to retrieve intermediate data, such as sequence, alignment, and phylogeny, is crucial to reproducing fungal taxonomic studies (Hibbett et al., 2016). Issues including human errors, the absence of intermediate information, and insufficient method descriptions (i.e. alignment editing, trimming criteria, and species range decision) have plagued fungal taxonomy, reducing the reproducibility of fungal phylogenetic analysis (Petersen et al., 2010; Shen et al., 2020). FunVIP addresses these challenges by consolidating all the step-by-step results into a single data directory. Researchers using FunVIP can easily deposit results as supplementary files, enabling others to reproduce the entire analysis with a single command.

Practical problems arising in fungal taxonomy were also considered in developing FunVIP. Large-scale insertions and fragmented sequences pose challenges in managing fungal sequence alignments. For instance, large-scale insertions are critical criteria for certain fungal species identification (Nagy et al., 2012). In the case of the genus *Trichoderma*, inconsistent transcription elongation factor regions are used by sequences (e.g. EU338335 contains intron 4 and 5 while DQ835489 includes exon 6), resulting in failing alignments (Dou et al., 2020; Rahimi et al., 2021). To address these alignment issues, FunVIP applies two solutions. Firstly, FunVIP uses the local alignment algorithm of MAFFT as default, instead of the widely used global alignment (Shen et al., 2021), enhancing performance with fragmented sequences or large-scale insertions (Thompson et al., 1999). Secondly, FunVIP preserves the non-terminal columns of the alignment, overcoming a limitation that may inadvertently remove phylogenetically important columns (Supple Data 3.1). Users can efficiently detect and manage such issues by combining these two solutions with the invalid alignment validation process (Supple Data 3.2).

Low-resolution genetic markers from closely related fungal species, such as the ITS region of *Penicillium* and *Aspergillus*, can produce polytomies in phylogenetic trees (Houbraken et al., 2021). Traditional tree-inferring tools, such as RAxML, only allow bifurcating trees, where the topology of the clades among identical sequences is not collapsed as intended. To overcome this, a polytomy-resolving algorithm and flat issue reporting system are integrated into FunVIP (Supple Data 8, Fig. S8.1). FunVIP reorganizes the topology of clades including zero-length branches based on annotation evidence. During these processes, FunVIP highlights issues that users should be cautious about. It is expected to facilitate a clearer interpretation of the phylogenetic tree when the genetic marker has low resolution.

For efficient tree inference, FunVIP employs the FastTree algorithm in the fast mode, replacing the commonly used neighbor-joining (NJ) method (Tamura et al., 2004). FastTree is recognized for balancing speed and accuracy, surpassing NJ methods (Price et al., 2010), and yielding results more closely coinciding with maximum likelihood (ML) methods like RAxML compared to the NJ method (Liu et al., 2011). Comparison of identification performance between FastTree (fast mode) and RAxML (accurate mode) with the *Sanghuangporus* and *Aspergillus* section *Terrei* datasets showed a small difference in accuracy, but a large decrease in time consumption (*Sanghuangporus*-fast: 56s, *Sanghuangporus*-accurate: 4,420s, *Aspergillus* section *Terrei*-fast: 55s, *Aspergillus* section *Terrei*-accurate: 4,767s). Therefore, running with FunVIP fast preset can be a good initial identification method, as it has a lower disparity with the ML method compared to the NJ method.

### Validation performance of FunVIP from the *Fuscoporia* dataset

Taxonomic reference data and sequences from public databases are considered as ground truth and used for species identification (Lücking et al., 2020a). However, mislabeled sequences are common in both public sequence databases (Hofstetter et al., 2019) and taxonomic reference data (Cho et al., 2023), leading to misidentifications (Lücking et al., 2020b). Exploring and interpreting all references used for the dataset can be difficult for users. Taxonomic experts can aid in curating these issues, but they are not always available. Therefore, FunVIP is designed to mimic the methods and expertise of taxonomists, to streamline and improve the accuracy of species identification.

Resolving mislabels is one of the most crucial steps in phylogeny-based identification using a public database containing misannotated sequences. One piece of evidence of mislabels is database conflicts: when samples within the database conflict with each other, it indicates that either of them is likely mislabeled. FunVIP detected 113 database conflicts in the *Fuscoporia* dataset, including taxonomic mislabels and minor conflicts. Most of the database conflicts found in the *Fuscoporia* dataset resulted from old names, orthographic variants, additional sequencing, and mistakes in uploading sequences to GenBank. Notably, these conflicts are a consequence of the rapid advancement of fungal taxonomy research (Dayarathne et al., 2016). FunVIP validated six name updates in subsequent references, such as *F. wahlbergii* re-identified as *F. australasica*, and four orthographic variants, such as *F. setifera* being misspelled as *F. setifer*. The other evidence to find mislabels is polyphyly. Multiple clades identified as the same species (polyphyly) usually do not make sense from an evolutionary point of view. Thus, polyphyly should be primarily checked by validating mislabeled samples (Kozlov et al., 2016; McDonald et al., 2012). FunVIP detected 108 instances polyphyly and provides a detailed evidence report to assist users in dataset validation by indicating “where to start” and “what to check”, thus offering a solution when taxonomic expertise is absent. In previous work by Kozlov et al. (2016), the Semi-Automatic Taxonomic Improvement and Validation Algorithm (SATIVA) employed a polyphyly-based approach to curate taxonomically mislabeled samples. SATIVA used the Evolutionary Placement Algorithm (EPA) (Berger et al., 2011) to address polyphyly issues. However, EPA may yield incorrect results if a significant portion of samples are mislabeled, a critical concern in fungal phylogeny [i. e. *F. gilva* from Cho et al. (2023)]. Additionally, the primary object of SATIVA is to curate databases used in metabarcoding methods. Therefore, some features required by the field of fungal taxonomy, such as full automation (SATIVA starts with alignment), multigene applicability, and identification ability, are not supported. Now, FunVIP meets the demand for a validation program similar to SATIVA in the field of fungal taxonomy.

FunVIP implements a sequence-set organization step to address extreme cases of mislabeled samples included in the database. The sequence-set organization step played a notable role in distinguishing mislabeled samples at the taxonomic group (genus) level. Typically, after taxonomic group designation with BLAST, only ingroup and outgroup samples are included for phylogenetic analysis (Dissanayake et al., 2020). However, FunVIP included ambiguous samples (*Fomes*, *Fomitiporia*, *Fulvifomes*, *Inonotus*, *Inocutis*, *Phellinopsis*, *Phellinidium*, *Phellinus*, and *Tropicoporus*) in the sequence-set when analysing the *Fuscoporia* dataset. This approach facilitated the detection of non-*Fuscoporia* samples incorrectly labeled as *Fuscoporia* samples, such as *F. coronadensis* (Fig. 3F).

As seen in the *Fuscoporia* example, the prevalence of taxonomically misidentified sequences in public databases presents a significant challenge, highlighting the need to correct these annotations to improve data quality used by the scientific community. Curating the annotations of these sequences offers a fundamental solution that benefits all researchers utilizing these resources. While GenBank and UNITE have systems in place to address major misidentifications (such as those at the phylum level), minor cases (such as those within the genus level) are either not open for correction in GenBank or manually curated through third-party annotation platforms like UNITE and PlutoF (Abarenkov et al., 2024). FunVIP facilitates the identification and confident evaluation of these minor misidentifications, streamlining the correction process. By combining FunVIP with third-party annotation systems, users can actively contribute their findings, enhancing the accuracy of public sequence databases. This collaborative effort is expected to significantly reduce errors across datasets, ultimately reducing the workload of the community.

### Identification performance of FunVIP compared with BLAST

NCBI BLAST is a widely used method for initial fungal identification. However, the identification accuracy of NCBI BLAST in our analysis ranged from 87.2% to 95.1%, which is too low to be confident. BLAST-based fungal identification has several limitations: susceptibility to mislabeled sequences in the database and inconsistencies in similarity cutoffs for delimiting fungal species (Johnson et al., 2008; Somervuo et al., 2016). In addition, the local alignment algorithm of BLAST can reflect only aligned region of the sequences, thus similarity criteria can lead to unexpected result (Kang et al., 2010; Nilsson et al., 2012). We implemented a validation ability in FunVIP that specifies potentially misannotated database samples based on polyphyletic clades. Furthermore, the species delimitation algorithm, which utilizes tree metrics (branch length and branch support), resolves issues related to inconsistent similarity cutoffs.

Inconsistencies in similarity cutoffs for delimiting fungal species, ranging from 0.93 to 1 in similarity scores in the ITS regions, pose a challenge (Vu et al., 2022). BLAST analysis exhibited significantly larger variations in accuracy, precision, recall, and F1 score, depending on the percent identity cutoff settings used (Supple Data 7). In contrast, FunVIP utilizes phylogenetic topology and branch length to capture the range of sequence variation for each species, which is reported as a better approach in cases involving mislabeled samples in the database (Ross et al., 2008). As a result, FunVIP demonstrates lower deviations in identification metrics, ensuring a more consistent outcome irrespective of user-applied options. Additionally, FunVIP can delimitate new species candidates, unlike NCBI BLAST and q2-feature-classifier.

### Limitations and prospects

FunVIP can validate potentially mislabeled data but cannot confirm which data is incorrect without additional information, such as geographical origin or morphological characteristics of the studied specimen or isolate. Moreover, decisions are often based on the type specimens, making species without a sequence from the type specimen problematic. Since FunVIP is designed as a pipeline for fungal identification, it may not cover all scenarios, such as manual alignment editing, genetic marker partitioning, and Bayesian analysis.

Although FunVIP was initially developed to address the specific challenges of accurate and efficient fungal identification, its theoretical adaptability extends beyond fungi. However, the algorithms were specifically tailored for fungal data —for instance, by preventing internal column trimming and reorganizing trees for low-resolution barcodes. Since the development of FunVIP was based on expertise in fungal systems, potential challenges specific to other taxa remain unaddressed. Expanding the usability of FunVIP as a universal tool requires rigorous testing by specialists in other taxa to identify and resolve these challenges. To improve user-friendliness, future versions of FunVIP are expected to incorporate additional features such as graphical user interfaces and web servers, enhancing the accessibility and versatility of the pipeline.

## Conclusion

Accurate sequence-based identification of fungal species is always in demand. We released FunVIP, a phylogeny-based validation and identification pipeline for fulfilling the demand in fungal identification. This pipeline offers distinctive features: automatic sequence download, detection of misannotated samples in the database, multi-genetic marker analysis, and interpretation of phylogenetic trees. Through three case studies using *Fuscoporia*, *Sanghuangporus*, and *Aspergillus* section *Terrei* datasets, FunVIP effectively validated mislabeled sequences from databases and demonstrated superior identification performance compared to the widely employed BLAST. Overall, FunVIP serves as a straightforward identification pipeline when a database is available, enhancing confidence in data accuracy and reducing the time required for iterative phylogenetic analysis.

## Figures and Tables

**Table 1.** Comparison of potential softwares for fungal identification

Software	Key features	Limitations in fungal identification	Intermediate result availability	Final identification result availability	Multiple genetic marker applicability	Reference
Phylogeny.fr	Customizable phylogeny-based identification	Requires user interpretation of tree outcomes	Yes	No	Yes	Dereeper et al. (2008)
One-click Fungal Phylogenetic Tool (OFPT)	Streamlined fungal phylogenetic analysis	Requires user interpretation of tree outcomes	Yes	No	Yes	Zeng et al. (2023)
SUPERSMART	Automated phylogeny-based analysis for established species	For species delimitation not identification	Yes	No	Yes	Antonelli et al. (2017)
OneTwoTree	Automated phylogeny-based analysis for established species	For species delimitation not identification	Yes	No	Yes	Drori et al. (2018)
SATIVA	Semi-automatic tool for validating phylogenetic trees	Prior phylogenetic analysis required	Yes	-	-	Kozlov et al. (2016)
q2-feature-classifier	QIIME 2 plugin for taxonomy classification using k-mer search	Limited to single marker sequences	No	Yes	No	Bokulich et al. (2018)
		Does not provide phylogenetic analysis or species identification				
BLAST	Local alignment-based similarity search against database	Limited to single marker sequences	No	No	Yes	Camacho et al. (2009)
		Does not provide phylogenetic analysis or species identification				
FunVIP	Fully automated phylogeny-based identification pipeline for fungi	Currently only tested with fungal datasets	Yes	Yes	Yes	This study

**Table 2.** Number of polyphyly issues and inconsistent issues reported in second FunVIP run with *Fuscoporia* dataset

Issue	Genetic marker	Criteria	Number of issues
Polyphyly	concatenated	samples	108
		clades	50
		species	20
	ITS	samples	109
		clades	48
		species	20
	LSU	samples	59
		clades	33
		species	14
	*RPB2*	samples	36
		clades	14
		species	7
	*TEF1*	samples	17
		clades	9
		species	6
Inconsistent			89

## Supplementary Information

Supple Data 1. Options applicable with FunVIP

Supple Data 2. Detailed information for sequence-set organization

Supple Data 2.1. Importance of proper sequence-set organization

Supple Data 2.2. Definition of sequence-set in FunVIP (Fig. S2.2)

Supple Data 2.3. Detailed processes and logics of sequence-set organization

Supple Data 2.4. Algorithm for suspicious database sequence sorting

Supple Data 2.5. Algorithm for outgroup database sequence sorting (Fig. S2.5)

Supple Data 2.6. Validation of linear regression-based BLAST bitscore interpolation

Supple Data 3. Detailed information about alignment issues

Supple Data 3.1. Example of inadvertent non-terminal column trimming caused by TrimAl.

Supple Data 3.2. Explanation and example of invalid alignments

Supple Data 4. Detailed algorithms for tree interpretation

Supple Data 5. Detailed processes for phylogenetic analysis of the *Fuscoporia* datasets

Supple Data 5.1. Dataset collection and initial curation

Supple Data 5.2. Iterative runs and curations

Supple Data 6. Detailed dataset preparation and evaluation method for the *Sanghuangporus* and *Aspergillus* section *Terrei* datasets.

Supple Data 6.1. *Sanghuangporus* dataset collection and curation

Supple Data 6.2. *Aspergillus* section *Terrei* dataset collection and curation

Supple Data 6.3. Evaluation of accuracy between FunVIP, BLAST and q2-feature-classifier

Supple Data 7. Mean and standard deviation values for accuracy, precision, recall, and F1 score of FunVIP, BLAST, and q2-feature-classifier using the *Sanghuangporus* and *Aspergillus* section *Terrei* datasets

Supple Data 8. Example of the polytomy-resolving process performed using FunVIP
