# Supplementary material for: FunVIP: Fungal Validation and Identification Pipeline based on phylogenetic analysis
Source: J Microbiol. 2025 Apr 29;63(4):e2411017. doi: 10.71150/jm.2411017 (PMC13577050; doi:10.71150/jm.2411017)
Supplement: Supplementary file 1 [file jm-2411017-Supplementary-Data-1.docx]

**Supple Data 1. Options applicable with FunVIP**

**FunVIP documentation**

usage: **FunVIP** [-h] [--query [QUERY ...]] [--db DB [DB ...]] [--gene [GENE ...]] [--email EMAIL ]
[--test TEST] [--thread THREAD] [--memory MEMORY] [--outdir OUTDIR] [--runname RUNNAME]
[--mode MODE] [--continue] [--step STEP] [--level LEVEL] [--all] [--confident CONFIDENT] [--search SEARCH] [--alignment ALIGNMENT] [--trim TRIM] [--modeltest MODELTEST] [--tree TREE]
[--bscutoff BSCUTOFF] [--highlight HIGHLIGHT] [--heightmultiplier HEIGHTMULTIPLIER]
[--maxwordlength MAXWORDLENGTH] [--backgroundcolor [BACKGROUNDCOLOR ...]]
[--outgroupcolor OUTGROUPCOLOR] [--ftype FTYPE] [--fsize FSIZE]
[--fsize_bootstrap FSIZE_BOOTSTRAP] [--verbose VERBOSE] [--maxoutgroup MAXOUTGROUP]
[--collapsedistcutoff COLLAPSEDISTCUTOFF] [--collapsebscutoff COLLAPSEBSCUTOFF]
[--bootstrap BOOTSTRAP] [--nosolveflat] [--regex [REGEX ...]] [--cluster-cutoff CLUSTER_CUTOFF]
[--cluster-evalue CLUSTER_EVALUE] [--cluster-wordsize CLUSTER_WORDSIZE]
[--mafft-algorithm MAFFT_ALGORITHM] [--mafft-op MAFFT_OP] [--mafft-ep MAFFT_EP]
[--trimAl-algorithm TRIMAL_ALGORITHM] [--trimAl-gt TRIMAL_GT] [--allow-innertrimming]
[--criterion CRITERION] [--noavx] [--outgroupoffset OUTGROUPOFFSET] [--nocachedb] [--usecache] [--tableformat TABLEFORMAT] [--nosearchresult] [--preset PRESET] [--version]

* If more than one flag is available, either of them can be applied (i.e. both --email and -e are available to use)

**-h, --help**: show this help message

**--email [EMAIL], -e [EMAIL]**: E-mail required to download data from GenBank (Without email, NCBI may ban your IP)

**--version**: Show version of FunVIP

**Main options**

**--query [QUERY ...], -q [QUERY ...]**:
Query FASTA or table (csv, tsv, or xlsx) files to analyze. You can either write file names (if they are in current directory), or file path (either full path or relative path is okay). See figure (LINK) to how to generate query files.

**--db [DB ...], -d DB [DB ...]** **<<REQUIRED!!!!>>**
Database table (csv, tsv, or xlsx) files. You can either write file names (if they are in current directory), or file path (either full path or relative path is okay). See figure (LINK) to how to generate database files.

**--gene [GENE ...], -g [GENE ...]** **<<REQUIRED!!!!>>**
Genetic marker names to be analyzed. Should be same to corresponding column name (Differences in capitalization are okay)

--**preset [fast] | [accurate] | [YAML_FORMAT_FILE]**

**fast**: Fast mode, use --auto for MAFFT and FastTree for tree construction.
**accurate**: Accurate mode, use --localpair for MAFFT and RAxML with 1000 rapid bootstrap for tree construction.
**YAML_FORMAT_FILE**: your custom set of options saved in yaml format. See <https://github.com/Changwanseo/FunVIP/tree/main/funvip/preset> to find out how to make it. Default: fast.

**Test run options**

**--test [Penicillium]** | **[Terrei]**
Use test dataset.
The Penicillium dataset compromises data from GenMine paper (Seo et al., 2022). It will take 10+ minutes in 12core system.
The Terrei dataset compromises data from the FunVIP paper (Seo et al., 2024, Unpublished). It will take 3+ minutes in 12c system. Terrei option requires additional email to also test downloading sequence from GenBank

**Running options**

**--thread [THREAD], -t [THREAD]**
Threads to be used for pipeline. Default: system maximum. If FunVIP uses too much memory, decreasing the thread count may help reduce memory usage.

**--memory [MEMORY], -m [MEMORY]**
Max memory limit in gigabytes. Write in 'nG' form, ex: '16G'. It should be more than 4G. Default: system maximum. This limit only applies for MMseqs and iqtree. Therefore, if FunVIP exceeds memory in other steps, please decrease thread number instead.

**--outdir [OUTDIR]**
Out file location, Default: current directory. If you run FunVIP with **--continue**, this option is mandatory to specify which previous run to use

**--runname [RUNNAME]**
Name prefix to current run. Default: current timestamp. If you run FunVIP with **--continue**, this option is mandatory to designate which previous run to use

**--mode [MODE]**
Mode setup in species identification, **[validation] | [identification].**
The mode decides species assignment of zero-length polytomy branch, if species name of the queries was given. For example, two database samples, *Penicillium* A, and *Penicillium* B show zero-length polytomy branch. In validation mode, if species name of the query is given as Penicillium A, FunVIP assigns query as Penicillium A (Trusts the query information) In identification mode, if query is given as Penicillium A, FunVIP assigns the query as Penicillium sp. 1, as it is not fully confident that the query belongs to Penicillium A or Penicillium B based on the query sequence alone.

**--continue**
Continue from previous run. Should be used with **--outdir**, **--runname**, and **--step** to specify the previous run directory and the steps to be continued

**--step** **[setup] | [search] | [cluster] | [align] | [trim] | [concatenate] | [modeltest] | [tree] | [visualize] | [report]**
Step to continue from previous run. Will be ignored without **--continue** option.
**[setup]**: Start from initial step, from input validation
**[search]**: Start from BLAST/MMseqs search among sequences
**[cluster]**: Start from taxonomic group assignment and outgroup selection **[align]**: Start from MAFFT alignment **[trim]**: Start from alignment trimming **[concatenate]**: Start from concatenating alignments **[modeltest]**: Start from model selection **[tree]**: Start from phylogenetic tree construction **[visualize]**: Start from tree interpretation and visualization **[report]**: Start from report

**--level [subseries] | [series] | [subsection] | [section] | [subtribe] | [subfamily] | [family] | [suborder] | [order] | [subclass] | [class] | [subphylum] | [phylum] | [subdivision] | [division] | [subkingdom] | [kingdom]**
Taxonomic level for each phylogenetic tree. Should be higher than the species level

**--all**
Run FunVIP for all database sequences, regardless of whether corrresponding sequences exist in the query. Default: False.
If no query sequences are provided, the **--all** flag will be automatically enabled.

**--maxoutgroup [MAXOUTGROUP**]
Maximum outgroup numbers to include in phylogenetic analysis, Default: 3. We recommend at least 3 to maxoutgroup to confirm quartet hypothesis.

**Method selection for each step of pipeline**

**--search [blast] | [mmseqs]**
Search methods used for selecting genetic markers, taxa and outgroups, Default: blast. BLAST is more sensitive, while mmseqs is faster in large number of sequences. MMseqs uses a large amount of memory, so more than 16GB or RAM is recommended

**--trim [trimAl] [gblocks] [none]**
Trimming methods. Default: trimAl. If **[none]** selected, trimming step will be skipped.
Default trimming methods are modified to avoid removing non-terminal columns. To use the original behavior of trimAl or Gblocks, use the **--allow-innertrimming** option

**--notcs**
Skip T-COFFEE TCS(Transitive Consistency Score) for alignment validation. default: False

**--modeltest** **[iqtree] [modeltestng] [none]**
Model selection methods. Default: none. If **[none]** selected, modeltest step will be skipped
Modeltest-ng is currently only available with Linux platform.

**--tree [fasttree] [iqtree] [raxml]**
Phylogenetic tree inference method. Default: fasttree

**Visualization options for drawing phylogenetic trees**

**--bscutoff [BSCUTOFF]**
Bootstrap cutoff for visualization. Default: 70. If 70, bootstrap values equals to or above 70 are represented in the tree

**--highlight [HIGHLIGHT]**
Color to highlight query sequences in tree visualization. Default: #AA0000 (Crimson like color). Either in html svg recognizable string or hex code, i.e. “red”, “blue”, “magenta” or “#03030A”.

**--heightmultiplier [HEIGHTMULTIPLIER]**
Height multiplier in drawing collapsed clades (triangles in the tree). Default: 6. A larger heightmultiplier expands the phylogenetic tree, while a smaller heightmultiplier reduces its height.

**--maxwordlength [MAXWORDLENGTH]**
Maximum number of letters to be shown in a single line of tree annotation. Default: 48.

**--backgroundcolor [BACKGROUNDCOLOR ...]**
List of background colors to be shown in tree, Default: “#f4f4f4” “#c6c6c6” (Very light pink and pink). Input should be enclosed in quotes, separated by spaces, and is recommended to in hex codes. To remove background, use --backgroundcolor "#FFFFFF" "#FFFFFF" (alternating white and white)

**--outgroupcolor [OUTGROUPCOLOR]**
Background color to indicate outgroup, default: #999999 (Dark gray). Use --outgroupcolor #FFFFFF to remove background color of outgroup.

**--ftype [FTYPE]**
Font for phylogenetic tree, default: Arial. The font should be installed in your computer, and some font may not work depending on your operating system.

**--fsize [FSIZE]**
Font size for the phylogenetic tree in points. Default: 10

**--fsize_bootstrap [FSIZE_BOOTSTRAP]**
Font size for bootstrap support in the phylogenetic tree in points. Default: 9

**Advanced options**

**--verbose [0] | [1] | [2] | [3], -v [0] | [1] | [2] | [3]**
Verbosity level to inform user and write in log file. Default: 2
0: error (Only inform when error occurs)
1: warning (Inform warnings and errors)
2: info (Default, inform info, warnings, and errors)
3: debug mode (Inform debug, info, warnings, and errors). Also changes multithreading behavior to check problems in FunVIP. If you find bugs that seem to be due to a deficiency of FunVIP, please run it again with **--verbose 3** and send all result files to the developer (via email or issue tabs in github repository).

**--collapsedistcutoff [COLLAPSEDISTCUTOFF**]
Species delimitation criteria according to phylogenetic distance. Samples within this tree distance of this value from the least common ancestor will be considered the same species. Default: 0.01 (1% differences from the least common ancestor are regarded as same species). This option does not deny topological evidence.

**--collapsebscutoff [COLLAPSEBSCUTOFF]**
Species delimitation criteria according to tree support percentage (bootstrap for IQTREE and RAxML, local support value for FastTree). Samples higher than this support of this value from the least common ancestor will be considered as same species. Default: 100 (Bootstrap is not used as species delimitation criteria). This option does not deny topological evidence.

**--bootstrap [BOOTSTRAP]**
Rapid boostrap number (RAxML) / Ultra-fast bootstrap number (IQTREE) for tree analysis. Will be ignored if fasttree is selected for tree method. Default: 1000. If you use IQTREE, bootstraps should be equal to or higher than 1000 to prevent errors.

**--nosolveflat**
Do not detect 0 length branch and automatically solve them, Default: False

**--regex [REGEX ...]**
Python regex list used to parse strain numbers from your input (see <https://docs.python.org/3/howto/regex.html>). This may be useful if your sequence descriptions are inconsistent or cluttered.
For example, if you put --regex "SFC[0-9]{8}-[0-9]{2}" for option, and your query looks like
“SFC20180902-01_Hymenochaetales_sp._ITS1.ab1”, only “SFC20180902-01” part will be parsed and used for final report.

**--cluster-cutoff [CLUSTER_CUTOFF]**
Minimum percent identity required to be considered the same taxonomic group in clustering analysis. Should be between 0 and 1.
Default: 0.97 (Over than 97% identity to database sequence from search result will be regarded as the taxonomic group). If your final tree includes too many unexpected queries, higher this value. If your final tree does not include expected query, lower this value (This rarely happens at genus level).

**--cluster-evalue [CLUSTER_EVALUE]**
E-value cutoffs for blast/mmseqs search. Values above this cutoff will be calculated and applied to the analysis. Default: 0.0000001

**--cluster-wordsize [CLUSTER_WORDSIZE]**
Word size for BLAST/MMseqs search. Default: 7

**--mafft-algorithm [localpair] | [globalpair] | [auto]**
MAFFT algorithm for alignment, see mafft documents.

**--mafft-op [MAFFT_OP]**
MAFFT gap opening penalty (op) value. Default: 1.3. It is highly recommended to increase this value to 2 or 3 when your alignment does not seem to be correct. You may have to use 5 in extreme cases.

**--mafft-ep [MAFFT_EP**]
MAFFT gap extension penalty (ep) value. Default: 0.1. If there are large gaps in your alignments, lowering this value may help.

**--trimAl-algorithm [gt] [gappyout] [strict] [strictplus]**
TrimAl algorithm for trimming. See the TrimAl documentation for details ([https://trimAl.readthedocs.io/en/latest/usage.html](https://trimal.readthedocs.io/en/latest/usage.html)).
Will be ignored if TrimAl is not selected for trimming option

**--trimAl-gt [TRIMAL_GT]**
Gap threshold (gt) value for TrimAl ([https://trimAl.readthedocs.io/en/latest/usage.html](https://trimal.readthedocs.io/en/latest/usage.html)). Default: 0.2. Will be ignored if TrimAl is not selected for trimming option

**--allow-innertrimming**
Turn off FunVIP’s adjustment that prevents trimming of non-terminal alignment columns. Default: False

**--criterion [AIC] [AICc] [BIC]**
Modeltest criterion to use, either AIC (Akaike’s Information Criterion), AICc (Akaike’s Information Criterion with a correction for a small design), or BIC (Bayesian information Criterion). The best matching model for the designated criterion will be used for tree inference.

**--noavx**
Do not use AVX acceleration for RAxML. Default: False. This will be automatically turned on if your system lacks the AVX instruction set.

**--outgroupoffset [OUTGROUPOFFSET]**
Outgroupoffset value in bitscore cutoff. Increasing this value will select a more distant outgroup, while decreasing this value will select a closer outgroup. If your outgroup is mixed with the ingroup in the phylogenetic tree, try increasing this value. Default: 20

**Saving options**

**--cachedb**
Disable caching current search database. Use it if your database is too big for system directory.
Default: True

**--usecache**
Use the cached search database. Turn off if your previously cached database is making error. Default: True

**--matrixformat** **[tsv] | [csv] | [xlsx]**
Default format for search matrix files. Default: csv

**--nosearchresult**
Do not save BLAST/MMseqs search matrix. Use this when the dataset becomes too large and generates IO bottleneck
