# Supplementary material for: FunVIP: Fungal Validation and Identification Pipeline based on phylogenetic analysis
Source: J Microbiol. 2025 Apr 29;63(4):e2411017. doi: 10.71150/jm.2411017 (PMC13577050; doi:10.71150/jm.2411017)
Supplement: Supplementary file 2 [file jm-2411017-Supplementary-Data-2-1.docx]

**Supple Data 2.1. Importance of proper sequence-set organization**

Careful taxon sampling is required to construct a high-resolution phylogenetic tree for precise identification (Hillis et al., 2003), however, fungal taxonomy is often challenged by inappropriate taxon sampling. A wide taxon sampling is defined as incorporating samples that may be phylogenetically far distant from the query (Fig. S2.1A). For example, in the case of *Fuscoporia* (*Hymenochaetales*), incloporating *Amanita* (*Agariacles*) reference sequences to the phylogenetic tree is a practice of a wide taxon sampling, since they belong to different orders. When a wide taxon sampling occurs, sequences are too distinct, and only a small number of sequences in the alignment are informative and account for the phylogenetic tree (Little, 2011; Lücking & Hawksworth, 2018). This ultimately results in a low-resolution phylogenetic tree (Fig. S2.1A).

Narrow taxon sampling is defined by incorporating only the phylogenetically closely related sequences (Fig. S2.1B). For example, in case of *Fuscoporia*, including only reference sequences of *Fuscoporia* to phylogenetic tree is an example for narrow taxon sampling. The issue with narrow taxon sampling is that user may be uncertain that the query truly belongs to the target genus of the study, in this case, *Fuscoporia*. In usual cases, the taxon of the unidentified sample is initially screened with NCBI BLAST. However, as there are many misidentified sequences in GenBank, NCBI BLAST may assign an incorrect taxon to the query. When narrow taxon sampling occurs, there are no comparable sequences to reveal that the taxonomic group assignment for the query is incorrect, which can ultimately lead to misidentification of the sequences (Fig. S2.1B). Therefore, proper sequence-set organization is important for phylogenetic analysis.

**Fig. S2.1.** Infographics illustrating the importance of proper sequence-set organization. (A) Example of wide sequence-set organization. (B) Example of narrow sequence-set organization.
