# Supplementary material for: FunVIP: Fungal Validation and Identification Pipeline based on phylogenetic analysis
Source: J Microbiol. 2025 Apr 29;63(4):e2411017. doi: 10.71150/jm.2411017 (PMC13577050; doi:10.71150/jm.2411017)
Supplement: Supplementary file 3 [file jm-2411017-Supplementary-Data-2-2.docx]

**Supple Data 2.2. Definition of sequence-set in FunVIP** (Fig. S2.2)

| ***Properties*** |  |  |
| --- | --- | --- |
| **Taxonomic group** | : | Specific taxa above species level for targeted analysis |
|  |  | e.g. *Fuscoporia* (genus), *Hymenochaetales* (order) |
| **Genetic marker** | : | Genetic element (including coding and non-coding regions) for analysis |
|  |  | e.g. ITS, LSU, *BenA*, *RPB2* |
|  |  |  |
| ***Contents*** |  |  |
| **Query** | : | Selected input sequence that is targeted for analysis |
| **Database** | : | Selected input sequence from a database consisting of **Ingroup**, **Suspicious**, and **Outgroup** sequences |
|  |  |  |
| **Ingroup** | : | **Database** sequence annotated as **Taxonomic group** of the sequence-set. |
|  |  | e.g. *Fuscoporia* sequence |
| **Suspicious** | : | **Database** sequence not annotated as belonging to the **Taxonomic group** but can potentially be an **Ingroup.** |
|  |  | e.g. *Fuscoporia* sequence annotated to *Sanghuangporus* |
| **Outgroup** | : | **Database** sequence that is confirmed not to belong to the **Taxonomic group**. |
|  |  | e.g. *Hydnoporia* sequence |

**Fig. S2.2.** Infographics illustrating the definition of sequence-sets in FunVIP. (A) Representation of the input query file and database file. (B) BLAST/MMseqs search space, where the proximity of sample pairs indicates a higher bitscore. (C) Visualization of the generated sequence-sets. (D) Key elements of the sequence-set and their respective roles in the final phylogenetic analysis.
