# Supplementary material for: FunVIP: Fungal Validation and Identification Pipeline based on phylogenetic analysis
Source: J Microbiol. 2025 Apr 29;63(4):e2411017. doi: 10.71150/jm.2411017 (PMC13577050; doi:10.71150/jm.2411017)
Supplement: Supplementary file 4 [file jm-2411017-Supplementary-Data-2-3.docx]

**Supple Data 2.3. Detailed processes and logics of sequence-set organization**

The sequence-set organization step is divided into two sub-steps: the “database sequence sorting step” and the “query sequence sorting step”. In the “database sequence sorting step”, FunVIP sort database sequences into each sequence-set from input database sequences. The “database sequence sorting step” is composed of three processes: i) “ingroup database sequence sorting”, ii) “suspicious database sequence sorting”, and iii) “outgroup database sequence sorting”. In the “ingroup database sequence sorting” process, database sequences annotated as belonging to the taxonomic group of the sequence-set (e.g., real *Fuscoporia* database sequence) are sorted to the sequence-set. In the “suspicious database sequence sorting” process, potential ingroup database sequences that are suspected to be misannotated as other taxa (e.g., *Fuscoporia* database sequence misannotated as “*Amanita*”) are sorted into the sequence-set. In the outgroup database sequence sorting step, database sequences confirmed to be from outside the target taxonomic group (e.g., *Sanghuangporus* database sequences as outgroup) are sorted to the sequence-set. Assigning all three types of database sequences ensures accurate tree interpretation, even in cases where mislabeled samples exist in the database (Supple Data 2.1, Fig. S2.1). The “query sequence sorting” step involves the assignment of each query to the user-specified taxonomic level (e.g., section, genus, or family, default: genus), and is followed by the closest BLAST or MMseqs2 match (Fig. S2.3A). When more than one genetic marker is provided for a sample, the sum of the bitscores is used to assign the taxa (Fig. S2.3B).

To compensate for any missing genetic markers, interpolation is performed using a linear regression model (Fig. S2.3C). A higher BLAST bitscore (hereafter, bitscore) between two sequences indicates a greater probability of a match, independent of sequence length and database size. Additionally, bitscore can be converted to -log E-value and is defined by *λ* × *S* – ln *K*/ln 2 (*S*: raw alignment score, *λ, K:* constants). The value *S* can be calculated as the sum of substitution and gap scores. Therefore, *S* can be approximately interpreted as the number of mutations per unit of sequence length. Molecular clocks can be generally considered to be proportional to bitscore. Conversely, each bitscore can be predicted through linear regression based on the estimated molecular clock. In detail, suppose a scenario involving a BLAST search between multiple genetic marker pairs of two strains. Evolutionary, these two strains diverged at a single point in time. Obtain *n* multiple genetic markers from each strain. For each genetic marker pair, bitscore can be calculated through BLAST. Let the bitscore of the *n*-th genetic marker pair *y_n_*. The equation (*c_n_ – y_n_*) */ k_n_ = K* holds for the bitscore constant *c_n_*, gradient *k_n_*, and time point constant *K* can be deduced using scipy.optimize.minimize module to minimize *R^2^* value of the linear regression. If the *k*-th genetic marker does not exist, the predicted bitscore *ŷ­_k_* can be the calculated using the equation *ŷ_k_* = *c_k_ -* $\bar{K}$ × *k_k_*, where $\bar{K}$ is calculated as the mean of *K­* predicted with *y*_i_, with i stands for index of genetic markers with bitscores available (Fig. S2.3C).

**Fig. S2.3.** Infographics illustrating the taxonomic group assignment method for a query sample. (A) Basic query taxonomic group assignment policy. (B) Query taxonomic group assignment using multiple genetic markers. (C) Query taxonomic group assignment using multiple genetic markers when certain genetic markers are absent.
