# Supplementary material for: FunVIP: Fungal Validation and Identification Pipeline based on phylogenetic analysis
Source: J Microbiol. 2025 Apr 29;63(4):e2411017. doi: 10.71150/jm.2411017 (PMC13577050; doi:10.71150/jm.2411017)
Supplement: Supplementary file 5 [file jm-2411017-Supplementary-Data-2-4.docx]

**Supple Data 2.4. Algorithm for suspicious database sequence sorting**

Suspicious sequences, which may be phylogenetically placed within the ingroup but are not annotated as such, are included in the sequence-set. This process is implemented to prevent potential misinterpretations of the phylogenetic tree that may arise from missing sequences (Fig. S2.5). We defined suspicious sequences as those that cannot be confidently classified as either ingroup or outgroup. Let *a_k_* denote an suspicious sequence, and *a* represent the taxonomic group of the suspicious sequence. The set of suspicious sequences, *a_k_* is determined using the following condition:

$$\{a_{k} |B_{i_{x}a_{k}}<\min\left( B_{i_{x}i_{y}} \right)-c \wedge\neg\max\left( B_{i_{x}a_{k}} \right)<\min\left( B_{i_{x}i_{y}} \right)-c\}$$

**Fig. S2.4.** Infographics illustrating the algorithm for suspicious database sequence sorting. (A) Assumed scenario for suspicious sequence inclusion. (B) Comparison of the effects when suspicious sequences are either included or excluded from the sequence set.
