# Supplementary material for: FunVIP: Fungal Validation and Identification Pipeline based on phylogenetic analysis
Source: J Microbiol. 2025 Apr 29;63(4):e2411017. doi: 10.71150/jm.2411017 (PMC13577050; doi:10.71150/jm.2411017)
Supplement: Supplementary file 6 [file jm-2411017-Supplementary-Data-2-5.docx]

**Supple Data 2.5. Algorithm for outgroup database sequence sorting** (Fig. S2.5)

The most appropriate outgroup, denoted as *t*, is automatically selected from the database. It is the closest group of sequences to the assigned taxonomic group, while still being distinctly different from assigned ingroup taxonomic group. Let *t_k_* represent the sequences of the outgroup taxonomic group, *i_x_* the sequence of the ingroup taxonomic group, *B_pq_* the bitscore value between sequences *p* and *q*, and *c* the outgroup offset. The outgroup taxonomic group *t* was chosen based on the following criterion:

$$t=\mathrm{argmin} \{min(B_{i_{x}t_{k}}) | max \left( B_{i_{x}t_{k}} \right)<\min\left( B_{i_{x}i_{y}} \right)-c\}$$

**Fig. S2.5.** Infographics illustrating the algorithm for outgroup database sequence sorting.
