# Supplementary material for: FunVIP: Fungal Validation and Identification Pipeline based on phylogenetic analysis
Source: J Microbiol. 2025 Apr 29;63(4):e2411017. doi: 10.71150/jm.2411017 (PMC13577050; doi:10.71150/jm.2411017)
Supplement: Supplementary file 7 [file jm-2411017-Supplementary-Data-2-6.docx]

**Supple Data 2.6** **Validation of linear regression-based BLAST bitscore interpolation**

We evaluated the interpolation of BLAST bitscore for missing genetic markers by randomly excluding and re-predicting genetic markers from a real dataset. A fungal kingdom-wide dataset comprising multiple genetic markers for 1,587 species and 61 genetic markers were obtained from the UFCG database (Kim et al., 2023). Species containing all four 4 commonly used genetic markers (*TUB2*, *RPB2*, *CMD1*, *TEF1*) were subsampled from the database, and species with genetic marker length under 90% threshold were excluded. Higher rank taxonomy (above the genus level) was replaced with a taxonomic system obtained from the UNITE database based on the original genus, as ambiguous taxonomic classifications were found in the UFCG dataset (e.g., the same genus assigned to different families). Genera not present in the UNITE database were filtered out, resulting in a final total of 1,007 species. Combinations of 1-3 genetic markers among the four genetic markers (totaling 14 combinations: 2^4^ [inclusion/exclusion choices to 4 genetic markers] - 2 [removing all 4 genetic markers, removing none]) were excluded, with 10 species assigned to each combination (totaling 140 species) designated as the query. Thus, 867 species remain in the database, while 140 species were designated as the query. The dataset division into database and query was conducted randomly, with adjustments made to ensure that species belonging to each genus were entirely removed. FunVIP runs were performed on both the species non-excluded version (UFCG) and the excluded version (UFCG_evaluate). The BLAST results from both datasets were parsed from each FunVIP run, and the prediction of BLAST bitscores was evaluated using Root Mean Squared Error (RMSE) metrics and R^2^ value for each genetic marker-by-genetic marker trendline. The analyses were conducted in 32-core, 64-thread Ryzen Threadripper platform with 512 GB of memory, utilizing --thread 16 option to address memory limitations.

The results of the interpolation of BLAST bitscores for missing genetic markers were visualized in the scatterplot (Fig. S2.6). RMSE values for each genetic marker were 115 (*TUB2*), 68 (*TEF1*), 296 (*RPB2*), and 30 (*CAM*), indicating small bitscore differences between the predicted and actual values. For the taxonomic assignment, sequences were accurately assigned at rates of 78.8% at the genus level, 85.6% at the family level and 90.4% at the order level. The UFCG follows the NCBI taxonomy as of 2019; thus, considering outdated misidentification and taxonomic errors within the UFCG taxonomy, the assignment performance can be utilized for approximate higher-level taxonomic assignments.

**Fig. S2.6.** Linear regression-based BLAST bitscore interpolation from the UFCG dataset. Scatterplot of bitscore versus bitscore values for each genetic marker pair. Predicted bitscores are indicated in color, while calculated bitscores are shown in gray. The linear regression model is represented by red dotted lines.

**Table S2.1.** Metrics for predicted bitscore values compared to calculated value. MAPE: Mean Absolute Percentage Error. MAE: Mean Absolute Error. RMSE: Root Mean Square Error. R^2^: Coefficient of determination from the regression line.

|  | MAPE | MAE | RMSE | MSE | R^2^ |
| --- | --- | --- | --- | --- | --- |
| TUB2 | 7.137689 | 36.37636 | 115.164 | 13262.74 | 0.91909 |
| TEF1 | 3.435564 | 25.3043 | 68.06914 | 4633.408 | 0.909765 |
| RPB2 | 53.23137 | 103.9907 | 295.8524 | 87528.64 | 0.880103 |
| CMD1 | 9.553155 | 10.15512 | 30.18846 | 911.3433 | 0.960698 |

**References**

Hillis DM, Pollock DD, McGuire JA, Zwickl DJ. 2003. Is sparse taxon sampling a problem for phylogenetic inference? *Syst Biol.* 52(1): 124.

Kim D, Gilchrist CL, Chun J, Steinegger M. 2023. UFCG: Database of universal fungal core genes and pipeline for genome-wide phylogenetic analysis of fungi. *Nucleic Acids Res.* 51(D1): D777–D784.

Little DP. 2011. DNA barcode sequence identification incorporating taxonomic hierarchy and within taxon variability. *PLoS One.* 6(8): e20552.

Lücking R, Hawksworth DL. 2018. Formal description of sequence-based voucherless fungi: Promises and pitfalls, and how to resolve them. *IMA Fungus.* 9(1): 143–165.
