# Supplementary material for: FunVIP: Fungal Validation and Identification Pipeline based on phylogenetic analysis
Source: J Microbiol. 2025 Apr 29;63(4):e2411017. doi: 10.71150/jm.2411017 (PMC13577050; doi:10.71150/jm.2411017)
Supplement: Supplementary file 8 [file jm-2411017-Supplementary-Data-2.docx]

**Supple Data 2. Detailed information for sequence-set organization**

**Supple Data 2.1. Importance of proper sequence-set organization**

Careful taxon sampling is required to construct a high-resolution phylogenetic tree for precise identification (Hillis et al., 2003), however, fungal taxonomy is often challenged by inappropriate taxon sampling. A wide taxon sampling is defined as incorporating samples that may be phylogenetically far distant from the query (Fig. S2.1A). For example, in the case of *Fuscoporia* (*Hymenochaetales*), incloporating *Amanita* (*Agariacles*) reference sequences to the phylogenetic tree is a practice of a wide taxon sampling, since they belong to different orders. When a wide taxon sampling occurs, sequences are too distinct, and only a small number of sequences in the alignment are informative and account for the phylogenetic tree (Little, 2011; Lücking & Hawksworth, 2018). This ultimately results in a low-resolution phylogenetic tree (Fig. S2.1A).

Narrow taxon sampling is defined by incorporating only the phylogenetically closely related sequences (Fig. S2.1B). For example, in case of *Fuscoporia*, including only reference sequences of *Fuscoporia* to phylogenetic tree is an example for narrow taxon sampling. The issue with narrow taxon sampling is that user may be uncertain that the query truly belongs to the target genus of the study, in this case, *Fuscoporia*. In usual cases, the taxon of the unidentified sample is initially screened with NCBI BLAST. However, as there are many misidentified sequences in GenBank, NCBI BLAST may assign an incorrect taxon to the query. When narrow taxon sampling occurs, there are no comparable sequences to reveal that the taxonomic group assignment for the query is incorrect, which can ultimately lead to misidentification of the sequences (Fig. S2.1B). Therefore, proper sequence-set organization is important for phylogenetic analysis.

**Fig. S2.1.** Infographics illustrating the importance of proper sequence-set organization. (A) Example of wide sequence-set organization. (B) Example of narrow sequence-set organization.

**Supple Data 2.2. Definition of sequence-set in FunVIP** (Fig. S2.2)

| ***Properties*** |  |  |
| --- | --- | --- |
| **Taxonomic group** | : | Specific taxa above species level for targeted analysis |
|  |  | e.g. *Fuscoporia* (genus), *Hymenochaetales* (order) |
| **Genetic marker** | : | Genetic element (including coding and non-coding regions) for analysis |
|  |  | e.g. ITS, LSU, *BenA*, *RPB2* |
|  |  |  |
| ***Contents*** |  |  |
| **Query** | : | Selected input sequence that is targeted for analysis |
| **Database** | : | Selected input sequence from a database consisting of **Ingroup**, **Suspicious**, and **Outgroup** sequences |
|  |  |  |
| **Ingroup** | : | **Database** sequence annotated as **Taxonomic group** of the sequence-set. |
|  |  | e.g. *Fuscoporia* sequence |
| **Suspicious** | : | **Database** sequence not annotated as belonging to the **Taxonomic group** but can potentially be an **Ingroup.** |
|  |  | e.g. *Fuscoporia* sequence annotated to *Sanghuangporus* |
| **Outgroup** | : | **Database** sequence that is confirmed not to belong to the **Taxonomic group**. |
|  |  | e.g. *Hydnoporia* sequence |

**Fig. S2.2.** Infographics illustrating the definition of sequence-sets in FunVIP. (A) Representation of the input query file and database file. (B) BLAST/MMseqs search space, where the proximity of sample pairs indicates a higher bitscore. (C) Visualization of the generated sequence-sets. (D) Key elements of the sequence-set and their respective roles in the final phylogenetic analysis.

**Supple Data 2.3. Detailed processes and logics of sequence-set organization**

The sequence-set organization step is divided into two sub-steps: the “database sequence sorting step” and the “query sequence sorting step”. In the “database sequence sorting step”, FunVIP sort database sequences into each sequence-set from input database sequences. The “database sequence sorting step” is composed of three processes: i) “ingroup database sequence sorting”, ii) “suspicious database sequence sorting”, and iii) “outgroup database sequence sorting”. In the “ingroup database sequence sorting” process, database sequences annotated as belonging to the taxonomic group of the sequence-set (e.g., real *Fuscoporia* database sequence) are sorted to the sequence-set. In the “suspicious database sequence sorting” process, potential ingroup database sequences that are suspected to be misannotated as other taxa (e.g., *Fuscoporia* database sequence misannotated as “*Amanita*”) are sorted into the sequence-set. In the outgroup database sequence sorting step, database sequences confirmed to be from outside the target taxonomic group (e.g., *Sanghuangporus* database sequences as outgroup) are sorted to the sequence-set. Assigning all three types of database sequences ensures accurate tree interpretation, even in cases where mislabeled samples exist in the database (Supple Data 2.1, Fig. S2.1). The “query sequence sorting” step involves the assignment of each query to the user-specified taxonomic level (e.g., section, genus, or family, default: genus), and is followed by the closest BLAST or MMseqs2 match (Fig. S2.3A). When more than one genetic marker is provided for a sample, the sum of the bitscores is used to assign the taxa (Fig. S2.3B).

To compensate for any missing genetic markers, interpolation is performed using a linear regression model (Fig. S2.3C). A higher BLAST bitscore (hereafter, bitscore) between two sequences indicates a greater probability of a match, independent of sequence length and database size. Additionally, bitscore can be converted to -log E-value and is defined by *λ* × *S* – ln *K*/ln 2 (*S*: raw alignment score, *λ, K:* constants). The value *S* can be calculated as the sum of substitution and gap scores. Therefore, *S* can be approximately interpreted as the number of mutations per unit of sequence length. Molecular clocks can be generally considered to be proportional to bitscore. Conversely, each bitscore can be predicted through linear regression based on the estimated molecular clock. In detail, suppose a scenario involving a BLAST search between multiple genetic marker pairs of two strains. Evolutionary, these two strains diverged at a single point in time. Obtain *n* multiple genetic markers from each strain. For each genetic marker pair, bitscore can be calculated through BLAST. Let the bitscore of the *n*-th genetic marker pair *y_n_*. The equation (*c_n_ – y_n_*) */ k_n_ = K* holds for the bitscore constant *c_n_*, gradient *k_n_*, and time point constant *K* can be deduced using scipy.optimize.minimize module to minimize *R^2^* value of the linear regression. If the *k*-th genetic marker does not exist, the predicted bitscore *ŷ­_k_* can be the calculated using the equation *ŷ_k_* = *c_k_ -* $\bar{K}$ × *k_k_*, where $\bar{K}$ is calculated as the mean of *K­* predicted with *y*_i_, with i stands for index of genetic markers with bitscores available (Fig. S2.3C).

**Fig. S2.3.** Infographics illustrating the taxonomic group assignment method for a query sample. (A) Basic query taxonomic group assignment policy. (B) Query taxonomic group assignment using multiple genetic markers. (C) Query taxonomic group assignment using multiple genetic markers when certain genetic markers are absent.

**Supple Data 2.4. Algorithm for suspicious database sequence sorting**

Suspicious sequences, which may be phylogenetically placed within the ingroup but are not annotated as such, are included in the sequence-set. This process is implemented to prevent potential misinterpretations of the phylogenetic tree that may arise from missing sequences (Fig. S2.5). We defined suspicious sequences as those that cannot be confidently classified as either ingroup or outgroup. Let *a_k_* denote an suspicious sequence, and *a* represent the taxonomic group of the suspicious sequence. The set of suspicious sequences, *a_k_* is determined using the following condition:

$$\{a_{k} |B_{i_{x}a_{k}}<\min\left( B_{i_{x}i_{y}} \right)-c \wedge\neg\max\left( B_{i_{x}a_{k}} \right)<\min\left( B_{i_{x}i_{y}} \right)-c\}$$

**Fig. S2.4.** Infographics illustrating the algorithm for suspicious database sequence sorting. (A) Assumed scenario for suspicious sequence inclusion. (B) Comparison of the effects when suspicious sequences are either included or excluded from the sequence set.

**Supple Data 2.5. Algorithm for outgroup database sequence sorting** (Fig. S2.5)

The most appropriate outgroup, denoted as *t*, is automatically selected from the database. It is the closest group of sequences to the assigned taxonomic group, while still being distinctly different from assigned ingroup taxonomic group. Let *t_k_* represent the sequences of the outgroup taxonomic group, *i_x_* the sequence of the ingroup taxonomic group, *B_pq_* the bitscore value between sequences *p* and *q*, and *c* the outgroup offset. The outgroup taxonomic group *t* was chosen based on the following criterion:

$$t=\mathrm{argmin} \{min(B_{i_{x}t_{k}}) | max \left( B_{i_{x}t_{k}} \right)<\min\left( B_{i_{x}i_{y}} \right)-c\}$$

**Fig. S2.5.** Infographics illustrating the algorithm for outgroup database sequence sorting.

**Supple Data 2.6** **Validation of linear regression-based BLAST bitscore interpolation**

We evaluated the interpolation of BLAST bitscore for missing genetic markers by randomly excluding and re-predicting genetic markers from a real dataset. A fungal kingdom-wide dataset comprising multiple genetic markers for 1,587 species and 61 genetic markers were obtained from the UFCG database (Kim et al., 2023). Species containing all four 4 commonly used genetic markers (*TUB2*, *RPB2*, *CMD1*, *TEF1*) were subsampled from the database, and species with genetic marker length under 90% threshold were excluded. Higher rank taxonomy (above the genus level) was replaced with a taxonomic system obtained from the UNITE database based on the original genus, as ambiguous taxonomic classifications were found in the UFCG dataset (e.g., the same genus assigned to different families). Genera not present in the UNITE database were filtered out, resulting in a final total of 1,007 species. Combinations of 1-3 genetic markers among the four genetic markers (totaling 14 combinations: 2^4^ [inclusion/exclusion choices to 4 genetic markers] - 2 [removing all 4 genetic markers, removing none]) were excluded, with 10 species assigned to each combination (totaling 140 species) designated as the query. Thus, 867 species remain in the database, while 140 species were designated as the query. The dataset division into database and query was conducted randomly, with adjustments made to ensure that species belonging to each genus were entirely removed. FunVIP runs were performed on both the species non-excluded version (UFCG) and the excluded version (UFCG_evaluate). The BLAST results from both datasets were parsed from each FunVIP run, and the prediction of BLAST bitscores was evaluated using Root Mean Squared Error (RMSE) metrics and R^2^ value for each genetic marker-by-genetic marker trendline. The analyses were conducted in 32-core, 64-thread Ryzen Threadripper platform with 512 GB of memory, utilizing --thread 16 option to address memory limitations.

The results of the interpolation of BLAST bitscores for missing genetic markers were visualized in the scatterplot (Fig. S2.6). RMSE values for each genetic marker were 115 (*TUB2*), 68 (*TEF1*), 296 (*RPB2*), and 30 (*CAM*), indicating small bitscore differences between the predicted and actual values. For the taxonomic assignment, sequences were accurately assigned at rates of 78.8% at the genus level, 85.6% at the family level and 90.4% at the order level. The UFCG follows the NCBI taxonomy as of 2019; thus, considering outdated misidentification and taxonomic errors within the UFCG taxonomy, the assignment performance can be utilized for approximate higher-level taxonomic assignments.

**Fig. S2.6.** Linear regression-based BLAST bitscore interpolation from the UFCG dataset. Scatterplot of bitscore versus bitscore values for each genetic marker pair. Predicted bitscores are indicated in color, while calculated bitscores are shown in gray. The linear regression model is represented by red dotted lines.

**Table S2.1.** Metrics for predicted bitscore values compared to calculated value. MAPE: Mean Absolute Percentage Error. MAE: Mean Absolute Error. RMSE: Root Mean Square Error. R^2^: Coefficient of determination from the regression line.

|  | MAPE | MAE | RMSE | MSE | R^2^ |
| --- | --- | --- | --- | --- | --- |
| TUB2 | 7.137689 | 36.37636 | 115.164 | 13262.74 | 0.91909 |
| TEF1 | 3.435564 | 25.3043 | 68.06914 | 4633.408 | 0.909765 |
| RPB2 | 53.23137 | 103.9907 | 295.8524 | 87528.64 | 0.880103 |
| CMD1 | 9.553155 | 10.15512 | 30.18846 | 911.3433 | 0.960698 |

**References**

Hillis DM, Pollock DD, McGuire JA, Zwickl DJ. 2003. Is sparse taxon sampling a problem for phylogenetic inference? *Syst Biol.* 52(1): 124.

Kim D, Gilchrist CL, Chun J, Steinegger M. 2023. UFCG: Database of universal fungal core genes and pipeline for genome-wide phylogenetic analysis of fungi. *Nucleic Acids Res.* 51(D1): D777–D784.

Little DP. 2011. DNA barcode sequence identification incorporating taxonomic hierarchy and within taxon variability. *PLoS One.* 6(8): e20552.

Lücking R, Hawksworth DL. 2018. Formal description of sequence-based voucherless fungi: Promises and pitfalls, and how to resolve them. *IMA Fungus.* 9(1): 143–165.
