# Supplementary material for: FunVIP: Fungal Validation and Identification Pipeline based on phylogenetic analysis
Source: J Microbiol. 2025 Apr 29;63(4):e2411017. doi: 10.71150/jm.2411017 (PMC13577050; doi:10.71150/jm.2411017)
Supplement: Supplementary file 9 [file jm-2411017-Supplementary-Data-3-1.docx]

**Supple Data 3.1. Example of inadvertent non-terminal column trimming caused by TrimAl.**

To evaluate the inadvertent removal of non-terminal columns from multiple sequence alignments by TrimAl, we compared four alignments: “Untrimmed”, “trimAl”, “trimAl + terminalonly”, and “FunVIP” (Fig. S3.1). FunVIP analysis of *Sanghuangporus* ITS sequences with accurate preset and distance cutoff 0.01 was selected as representative. “Untrimmed” and “FunVIP” alignments are parsed from original FunVIP analysis. Alignments of “trimAl” and “trimAl + terminalonly” are generated by running trimAl against “Untrimmed” alignment with and without the --terminalonly option respectively. To compare result of four alignments, “Wu 1805-2” sequences from each of the alignments were selected and realigned with MAFFT v 7.308 “auto” algorithm with 200 PAM / κ=2 scoring matrix with gap open penalty 3 and offset value 0 in Geneious 9.1.8 software (https://www.geneious.com). As a result of comparing the sequences from each alignment, we found that some bases in non-termianal columns were removed despite the --terminalonly option applied to trimAl, which appears unintended (Fig. S3.1).

**Fig. S3.1.** Example of inadvertent non-terminal column trimming caused by trimAl. Blue lines at each end indicate terminal trimming sites of the alignment, and regions labeled 1 – 9 refers inadvertently trimmed non-terminal columns. Red regions (1 – 5, 8, 9) show trimmed non-terminal columns despite the application of the --terminalonly option in trimAl. Blue regions (6, 7) represent trimmed non-terminal columns with the default trimAl option, but these were resolved when the --terminalonly option was applied.
