# Supplementary material for: FunVIP: Fungal Validation and Identification Pipeline based on phylogenetic analysis
Source: J Microbiol. 2025 Apr 29;63(4):e2411017. doi: 10.71150/jm.2411017 (PMC13577050; doi:10.71150/jm.2411017)
Supplement: Supplementary file 10 [file jm-2411017-Supplementary-Data-3-2.docx]

**Supple Data 3.2. Explanation and example of invalid alignments**

We define an invalid alignment as one without any column entirely composed of invalid bases (other than A, T, C, or G) for all sequences. Mathematically, we assume that each aligned sequence is represented as a vector, with bases substituted by 1 for valid bases (A, T, C, or G) and 0 for others (e.g., N, gaps, or IUPAC ambiguous letters). If the inner product of all vectors results in a zero vector, the alignment is considered invalid. These alignments are defined invalid because incomparable sequence pair exists in the alignment (Fig. S3.2). Invalid alignments are typically produced when different regions of the same genetic marker are used.

**Fig. S3.2.** Example of an invalid alignment. Sequence 2 and 3 do not share any comparable base pairs, and thus, the given alignment is considered invalid.
