# Supplementary material for: FunVIP: Fungal Validation and Identification Pipeline based on phylogenetic analysis
Source: J Microbiol. 2025 Apr 29;63(4):e2411017. doi: 10.71150/jm.2411017 (PMC13577050; doi:10.71150/jm.2411017)
Supplement: Supplementary file 11 [file jm-2411017-Supplementary-Data-3.docx]

**Supple Data 3. Detailed information about alignment issues**

**Supple Data 3.1. Example of inadvertent non-terminal column trimming caused by TrimAl.**

To evaluate the inadvertent removal of non-terminal columns from multiple sequence alignments by TrimAl, we compared four alignments: “Untrimmed”, “trimAl”, “trimAl + terminalonly”, and “FunVIP” (Fig. S3.1). FunVIP analysis of *Sanghuangporus* ITS sequences with accurate preset and distance cutoff 0.01 was selected as representative. “Untrimmed” and “FunVIP” alignments are parsed from original FunVIP analysis. Alignments of “trimAl” and “trimAl + terminalonly” are generated by running trimAl against “Untrimmed” alignment with and without the --terminalonly option respectively. To compare result of four alignments, “Wu 1805-2” sequences from each of the alignments were selected and realigned with MAFFT v 7.308 “auto” algorithm with 200 PAM / κ=2 scoring matrix with gap open penalty 3 and offset value 0 in Geneious 9.1.8 software (https://www.geneious.com). As a result of comparing the sequences from each alignment, we found that some bases in non-termianal columns were removed despite the --terminalonly option applied to trimAl, which appears unintended (Fig. S3.1).

**Fig. S3.1.** Example of inadvertent non-terminal column trimming caused by trimAl. Blue lines at each end indicate terminal trimming sites of the alignment, and regions labeled 1 – 9 refers inadvertently trimmed non-terminal columns. Red regions (1 – 5, 8, 9) show trimmed non-terminal columns despite the application of the --terminalonly option in trimAl. Blue regions (6, 7) represent trimmed non-terminal columns with the default trimAl option, but these were resolved when the --terminalonly option was applied.

**Supple Data 3.2. Explanation and example of invalid alignments**

We define an invalid alignment as one without any column entirely composed of invalid bases (other than A, T, C, or G) for all sequences. Mathematically, we assume that each aligned sequence is represented as a vector, with bases substituted by 1 for valid bases (A, T, C, or G) and 0 for others (e.g., N, gaps, or IUPAC ambiguous letters). If the inner product of all vectors results in a zero vector, the alignment is considered invalid. These alignments are defined invalid because incomparable sequence pair exists in the alignment (Fig. S3.2). Invalid alignments are typically produced when different regions of the same genetic marker are used.

**Fig. S3.2.** Example of an invalid alignment. Sequence 2 and 3 do not share any comparable base pairs, and thus, the given alignment is considered invalid.
