# Supplementary material for: FunVIP: Fungal Validation and Identification Pipeline based on phylogenetic analysis
Source: J Microbiol. 2025 Apr 29;63(4):e2411017. doi: 10.71150/jm.2411017 (PMC13577050; doi:10.71150/jm.2411017)
Supplement: Supplementary file 12 [file jm-2411017-Supplementary-Data-4.docx]

**Supple Data 4. Detailed algorithms for tree interpretation**

Initially, the phylogenetic tree is rooted using the outgroup selected during the dataset generation step (Fig. S4.1A). Subsequently, monophyletic taxa are identified based on taxonomic annotation and tree topology (Fig. S4.1B). Simultaneously, polytomies are recognized based on zero-length branches and resolved by rearranging the nodes according to taxonomic information. In the third step, paraphyletic query sequences are either merged with the neighboring monophyletic clade or separated based on branch length and bootstrap cutoffs (optional) (Fig. S4.1C). Separated clades without database sequences (containing only query sequences) are considered new species candidates, and novel species identifiers (e.g., “1” for “sp. 1”) are then assigned. Multiple monophyletic clades annotated with the same taxon name (hereafter, polyphyly) are numbered to inform users to check for potential taxonomically mislabeled samples in the database (Fig. S4.1D). Next, novel species identifiers are synchronized between single-locus and concatenated trees (Fig. S4.1E). The monophyletic clades in each genetic marker tree are either merged or separated based on the concatenated tree. Finally, FunVIP generates a report in a table format for the identification result and all potential issues detected during the pipeline run and visualizes phylogenetic trees in a vector image format suitable for further editing (Fig. S4.1F).

**Fig. S4.1.** Tree interpretation algorithms performed in FunVIP. Infographics describing algorithms performed during the tree interpretation step. Taxa α, β, and γ indicate database samples, O indicates database samples selected as the outgroup, and x, y, and z indicate query samples. Red, blue, and green colors indicate taxa α, β, and γ respectively. (A) Rooting of outgroup. Crimson squares indicate the outgroup. (B) Polytomy-resolving and monophyly detection. The crimson square indicates polytomy of taxon γ, and dotted squares indicate species’ borders defined by monophyly detection. The bidirectional arrow indicates recursive calls between the two functions. (C) Metric (tree distance, bootstrap)-based clustering. Dotted squares indicate species’ borders adjusted by metric-based clustering. MRCA stands for the Most Recent Common Ancestor. (D) Collapsing. (E) Synchronizing across each genetic marker tree. The crimson square indicates different criteria applied for collapsing by each genetic marker tree. Purple circles indicate sample z assigned to different species by each genetic marker tree. (F) Visualizing and reporting.
