# Supplementary material for: FunVIP: Fungal Validation and Identification Pipeline based on phylogenetic analysis
Source: J Microbiol. 2025 Apr 29;63(4):e2411017. doi: 10.71150/jm.2411017 (PMC13577050; doi:10.71150/jm.2411017)
Supplement: Supplementary file 13 [file jm-2411017-Supplementary-Data-5-1.docx]

**Supple Data 5.1. Dataset collection and initial curation**

A total of seven references mentioned in table 1 of Cho et al. (2023) were utilized for database preparation (Brazee, 2015; Chen & Dai, 2019; Chen & Yuan, 2017; Chen et al., 2020; Du et al., 2020, Tchoumi et al., 2020; Vlasak et al., 2020). Sequences from Cho et al., labeled as “This study” were obtained directly from the *IMA Fungus* website and used as a query. Tables from references published in *Mycokeys* and *Mycologia* were directly parsed from the respective websites, while tables from references published in *Mycosphere* and *Phytotaxa* were extracted using ABBYY FineReader PDF (https://pdf.abbyy.com/) to prepare a database file. With Microsoft Excel, “genus / species” columns were split into separate “genus” column and “species” columns. The “Voucher” column was renamed to “ID”. Columns such as “nLSU” and “nrLSU” were unified under “LSU” for consistency. Also, subscripts following accession numbers (e.g., the asterisk in GenBank accession “XX000000*”) were removed. The “Genus” and “Species” columns in the query file were removed to reproduce the situation of the original study, where the query sequences had not yet been identified.
