# Supplementary material for: FunVIP: Fungal Validation and Identification Pipeline based on phylogenetic analysis
Source: J Microbiol. 2025 Apr 29;63(4):e2411017. doi: 10.71150/jm.2411017 (PMC13577050; doi:10.71150/jm.2411017)
Supplement: Supplementary file 14 [file jm-2411017-Supplementary-Data-5-2.docx]

**Supple Data 5.2. Iterative runs and curations**

*** Note: See “~~~~~” refers to do designated file in the archived raw data of the article, as indicated in the ACKNOWLEDGEMENT (**https://doi.org/[10.5281/zenodo.14650006](https://zenodo.org/doi/10.5281/zenodo.13147233)**)**

The first run (run1) of FunVIP was conducted using fast mode. The run aborted with a total of 113+1 errors (See 300_case_study/run1/run1/log.txt). The additional error (indicated by “+1” in “133+1”) serves to inform users that errors occurred during the FunVIP run, resulting in termination. The 113 errors were caused by database conflicts, including species and sequence conflicts. Ten species conflicts were caused by six name updates in subsequent reference (one *F. ambigua* was re-identified as *F. ferruginosa*, one *F. gilva* was reidentified as *F. chinensis*, two *F.* *ferruginosa* were re-identified as *F. ambigua* and two *F. wahlbergii* were re-identified as *F. australasica*) and four orthographic variants (i.e., *F. setifera* was mistakenly typed as *F. setifer*, *F. septiseta* was mistakenly typed as *F. septoseta*). The remaining 103 sequence conflicts consisted of two partial sequences uploaded in different samples and 101 cases where sequence were added later (FunVIP detects conflict even when one of the sequences is blank). These database conflicts were resolved, and consequent FunVIP runs were conducted.

The second run (run2) was executed in fast mode. The number of inconsistent issues and polyphyly issues reported in each iteration of the run are described (Supple Table 5.1). The automatically visualized concatenated tree from the second run (see 300_case_study/run2/run2/07_Tree/run2_Fuscoporia_concatenated.svg) revealed seven samples with long-branch problems and one branch with a taxonomically mislabeled sample at the genus level. To investigate the causes of the long-branch problem, the alignment file (see 300_case_study/run2/run2/05_alignment/run2_trimmed_Fuscoporia_concatenated.fasta) was examined using Geneious 9.1.8 software with the “sort by alignment length” function. As a result, the ITS and LSU sequences were interchangeably placed, and this was detected based on NCBI BLAST analysis.

**References**

Chen Q, Dai YC. 2019. Two new species of *Fuscoporia* (*Hymenochaetales*, *Basidiomycota*) from southern China based on morphological characters and molecular evidence. *MycoKeys.* 61: 75.

Chen Q, Du P, Vlasák J, Wu F, Dai YC. 2020. Global diversity and phylogeny of *Fuscoporia* (*Hymenochaetales*, *Basidiomycota*). *Mycosphere.* 11(1): 1477–1513.

Chen Q, Wu F, Ji XH, Si J, Zhou LW, et al. 2019. Phylogeny of the genus *Fuscoporia* and taxonomic assessment of the *F. contigua* group. *Mycologia.* 111(3): 423–444.

Chen Q, Yuan Y. 2017. A new species of *Fuscoporia* (*Hymenochaetales*, *Basidiomycota*) from southern China. *Mycosphere.* 8(6): 1238–1245.

Cho Y, Kim D, Lee Y, Jeong J, Hussain S, et al. 2023. Validation of *Fuscoporia* (*Hymenochaetales*, *Basidiomycota*) ITS sequences and five new species based on multi-marker phylogenetic and morphological analyses. *IMA Fungus.* 14(1): 12.

Du P, Chen Q, Vlasák J. 2020. *Fuscoporia ambigua* sp. nov., a new species from America and China. *Phytotaxa.* 456(2): 175–185.

Tchoumi JM, Coetzee MP, Rajchenberg M, Roux J. 2020. Poroid *Hymenochaetaceae* associated with trees showing wood-rot symptoms in the Garden Route National Park of South Africa. *Mycologia.* 112(4): 722–741.

Vlasák J, Kout J, Chen Q, Dai YC. 2020. *Fuscoporia caymanensis* sp. nov. (*Basidiomycota*, *Hymenochaetaceae*), a new species from tropical America. *Phytotaxa.* 472(2): 135–146.
