# Supplementary material for: FunVIP: Fungal Validation and Identification Pipeline based on phylogenetic analysis
Source: J Microbiol. 2025 Apr 29;63(4):e2411017. doi: 10.71150/jm.2411017 (PMC13577050; doi:10.71150/jm.2411017)
Supplement: Supplementary file 16 [file jm-2411017-Supplementary-Data-6-1.docx]

**Supple Data 6.1. *Sanghuangporus* dataset collection and curation**

The Table 1 of the referenced research article by Shen et al. (2021) was downloaded using Camelot PDF table extractor (https://github.com/camelot-dev/camelot) and manually verified to ensure the parsed table and original PDF file indicate the same data. For the FunVIP database, ITS sequences indicated with term “Type” or “Reliable” from the “TAXONOMY” section were selected (n=15), and the remaining samples in the table were used as queries (n=256). We included additional *Tropicoporus* sequences (n=2, *Tropicoporus angustisculcantus* BJFC 024943 and *Tropicoporus excentrodendri* Yuan 6227) from order level study (Wu et al. 2022) for outgroup sequences to fulfill the outgroup sequence requirement of FunVIP. ITS sequences, MT343579 of Dai 13360 (BJFC) and MT343580 of Yuan 6438 (IFP) were misspellings of MT348579 and MT348580, respectively, and were detected during the pre-analysis of the data (See 900_evidence_for_editing/Sanghuangporus_pre/). Two non-*Sanghuangporus* samples (WN-3, DL 101) were removed because corresponding database sequences for those samples did not exist in the database. We incorporated these changes into analysis. The column “Species name accepted here” in the table is regarded as the ground truth identification.
