# Supplementary material for: FunVIP: Fungal Validation and Identification Pipeline based on phylogenetic analysis
Source: J Microbiol. 2025 Apr 29;63(4):e2411017. doi: 10.71150/jm.2411017 (PMC13577050; doi:10.71150/jm.2411017)
Supplement: Supplementary file 17 [file jm-2411017-Supplementary-Data-6-2.docx]

**Supple Data 6.2. *Aspergillus* section *Terrei* dataset collection and curation**

The query URM strains included in “Table 1” of the original research were initially parsed to CSV format using Camelot and manually verified to ensure that the parsed table and the original PDF indicated the same data (Barros et al. 2020). The column “Current name” was regarded as the ground truth identification. The database strains were parsed using the same method as the original research by manually locating GenBank accession numbers corresponding to strain numbers in “Figure 1” from the referenced research (Arzanlou et al., 2016; Guinea et al., 2015; Huang et al., 2020; Hubka et al., 2015; Samson et al., 2011, 2014; Visagie & Houbraken, 2020). Four of these were orthgraphic variants, with the author having misspelled *A. hortai* as *A. hortae.* The single sample (CMV0047) was annotated as *A.* cf. *alabamensis* in the table but annotated as *A*. *alabamensis* in phylogenetic tree. We followed the annotation provided in the phylogenetic tree for this sample. The authors also noted that *A. aureoterreus* CBS 503.65 was reidentified as *A. recifensis* in their paper (Barros et al., 2020), we also applied this adjustment to the database.
