# Supplementary material for: FunVIP: Fungal Validation and Identification Pipeline based on phylogenetic analysis
Source: J Microbiol. 2025 Apr 29;63(4):e2411017. doi: 10.71150/jm.2411017 (PMC13577050; doi:10.71150/jm.2411017)
Supplement: Supplementary file 18 [file jm-2411017-Supplementary-Data-6-3.docx]

**Supple Data 6.3. Evaluation of accuracy between FunVIP, BLAST and q2-feature-classifier**

For FunVIP, combinations of the --preset (fast, accurate) and the --collapsedistcutoff (0.005, 0.01, 0.02, 0.03, 0.05, 0.1, 0.25, and 0.5) options were used to evaluate metrics. BLAST does not provide identification criteria, we used cutoffs of 99.5, 99, 98, 97, 95, 75 and 50 for percent identity to calculate whether to assign species of the query to matched database species. Macro-averaged accuracy (https://scikit-learn.org/stable/modules/generated/sklearn.metrics.average_precision_score.html), precision, recall (https://scikit-learn.org/stable/modules/generated/sklearn.metrics.precision_recall_fscore_support.html), and F1 scores (https://scikit-learn.org/stable/modules/generated/sklearn.metrics.f1_score.html) were calculated as metrics for the multi-class classifier. Positives were counted if the species were well assigned to given database species or as a new species. Owing to the limitations of BLAST and q2-feature-classifier, delimitation of individual new species (e.g., sp. 1 and sp. 2) was not accounted for in the statistics (all regarded as “sp.”). Additionally, FunVIP accurate mode consumes a lot of time during phylogenetic tree construction due to the high computational demand of RAxML. Therefore, iterative analysis for statistics with varied parameters were performed using the --continue and --step visualization flags.

**References**

Arzanlou M, Samadi R, Frisvad JC, Houbraken J, Ghosta Y. 2016. Two novel *Aspergillus* species from hypersaline soils of the national park of Lake Urmia, Iran. *Mycol Prog.* 15(10): 1081–1092.

Barros Correia AC, Barbosa RN, Frisvad JC, Houbraken J, Souza-Motta CM. 2020. The polyphasic re-identification of a Brazilian *Aspergillus* section *Terrei* collection led to the discovery of two new species. *Mycol Prog.* 19: 885–903.

Guinea J, Sandoval-Denis M, Escribano P, Peláez T, Guarro J, et al. 2015. *Aspergillus citrinoterreus*, a new species of section *Terrei* isolated from samples of patients with nonhematological predisposing conditions. *J Clin Microbiol.* 53(2): 611–617.

Huang P, Jiang X, Wu B, Sun J. 2020. *Aspergillus jilinensis* sp. nov. and its thermostable alkaline enzymes evaluation. *Mycoscience.* 61(5): 205–211.

Hubka V, Nováková A, Kolařík M, Jurjević Ž, Peterson SW. 2015. Revision of *Aspergillus* section *Flavipedes*: Seven new species and proposal of section *Jani* sect. nov. *Mycologia.* 107(1): 169–208.

Samson RA, Peterson SW, Frisvad JC, Varga J. 2011. New species in *Aspergillus* section *Terrei*. *Stud Mycol.* 69(1): 39–55.

Samson RA, Visagie CM, Houbraken J, Hong SB, Hubka V, et al. 2014. Phylogeny, identification and nomenclature of the genus *Aspergillus*. *Stud Mycol.* 78(1): 141–173.

Shen S, Liu SL, Jiang JH, Zhou LW. 2021. Addressing widespread misidentifications of traditional medicinal mushrooms in *Sanghuangporus* (Basidiomycota) through ITS barcoding and designation of reference sequences. *IMA Fungus.* 12(1).

Visagie CM, Houbraken J. 2020. Updating the taxonomy of *Aspergillus* in South Africa. *Stud Mycol.* 95(1): 253–292.

Wu F, Zhou LW, Vlasák J, Dai YC. 2022. Global diversity and systematics of *Hymenochaetaceae* with poroid hymenophore. *Fungal Divers.* 113(1): 1–192.
