# Supplementary material for: FunVIP: Fungal Validation and Identification Pipeline based on phylogenetic analysis
Source: J Microbiol. 2025 Apr 29;63(4):e2411017. doi: 10.71150/jm.2411017 (PMC13577050; doi:10.71150/jm.2411017)
Supplement: Supplementary file 19 [file jm-2411017-Supplementary-Data-6.docx]

**Supple Data 6. Detailed dataset preparation and evaluation method for the *Sanghuangporus* and *Aspergillus* section *Terrei* datasets.**

**Supple Data 6.1. *Sanghuangporus* dataset collection and curation**

The Table 1 of the referenced research article by Shen et al. (2021) was downloaded using Camelot PDF table extractor (https://github.com/camelot-dev/camelot) and manually verified to ensure the parsed table and original PDF file indicate the same data. For the FunVIP database, ITS sequences indicated with term “Type” or “Reliable” from the “TAXONOMY” section were selected (n=15), and the remaining samples in the table were used as queries (n=256). We included additional *Tropicoporus* sequences (n=2, *Tropicoporus angustisculcantus* BJFC 024943 and *Tropicoporus excentrodendri* Yuan 6227) from order level study (Wu et al. 2022) for outgroup sequences to fulfill the outgroup sequence requirement of FunVIP. ITS sequences, MT343579 of Dai 13360 (BJFC) and MT343580 of Yuan 6438 (IFP) were misspellings of MT348579 and MT348580, respectively, and were detected during the pre-analysis of the data (See 900_evidence_for_editing/Sanghuangporus_pre/). Two non-*Sanghuangporus* samples (WN-3, DL 101) were removed because corresponding database sequences for those samples did not exist in the database. We incorporated these changes into analysis. The column “Species name accepted here” in the table is regarded as the ground truth identification.

**Supple Data 6.2. *Aspergillus* section *Terrei* dataset collection and curation**

The query URM strains included in “Table 1” of the original research were initially parsed to CSV format using Camelot and manually verified to ensure that the parsed table and the original PDF indicated the same data (Barros et al. 2020). The column “Current name” was regarded as the ground truth identification. The database strains were parsed using the same method as the original research by manually locating GenBank accession numbers corresponding to strain numbers in “Figure 1” from the referenced research (Arzanlou et al., 2016; Guinea et al., 2015; Huang et al., 2020; Hubka et al., 2015; Samson et al., 2011, 2014; Visagie & Houbraken, 2020). Four of these were orthgraphic variants, with the author having misspelled *A. hortai* as *A. hortae.* The single sample (CMV0047) was annotated as *A.* cf. *alabamensis* in the table but annotated as *A*. *alabamensis* in phylogenetic tree. We followed the annotation provided in the phylogenetic tree for this sample. The authors also noted that *A. aureoterreus* CBS 503.65 was reidentified as *A. recifensis* in their paper (Barros et al., 2020), we also applied this adjustment to the database.

**Supple Data 6.3. Evaluation of accuracy between FunVIP, BLAST and q2-feature-classifier**

For FunVIP, combinations of the --preset (fast, accurate) and the --collapsedistcutoff (0.005, 0.01, 0.02, 0.03, 0.05, 0.1, 0.25, and 0.5) options were used to evaluate metrics. BLAST does not provide identification criteria, we used cutoffs of 99.5, 99, 98, 97, 95, 75 and 50 for percent identity to calculate whether to assign species of the query to matched database species. Macro-averaged accuracy (https://scikit-learn.org/stable/modules/generated/sklearn.metrics.average_precision_score.html), precision, recall (https://scikit-learn.org/stable/modules/generated/sklearn.metrics.precision_recall_fscore_support.html), and F1 scores (https://scikit-learn.org/stable/modules/generated/sklearn.metrics.f1_score.html) were calculated as metrics for the multi-class classifier. Positives were counted if the species were well assigned to given database species or as a new species. Owing to the limitations of BLAST and q2-feature-classifier, delimitation of individual new species (e.g., sp. 1 and sp. 2) was not accounted for in the statistics (all regarded as “sp.”). Additionally, FunVIP accurate mode consumes a lot of time during phylogenetic tree construction due to the high computational demand of RAxML. Therefore, iterative analysis for statistics with varied parameters were performed using the --continue and --step visualization flags.

**References**

Arzanlou M, Samadi R, Frisvad JC, Houbraken J, Ghosta Y. 2016. Two novel *Aspergillus* species from hypersaline soils of the national park of Lake Urmia, Iran. *Mycol Prog.* 15(10): 1081–1092.

Barros Correia AC, Barbosa RN, Frisvad JC, Houbraken J, Souza-Motta CM. 2020. The polyphasic re-identification of a Brazilian *Aspergillus* section *Terrei* collection led to the discovery of two new species. *Mycol Prog.* 19: 885–903.

Guinea J, Sandoval-Denis M, Escribano P, Peláez T, Guarro J, et al. 2015. *Aspergillus citrinoterreus*, a new species of section *Terrei* isolated from samples of patients with nonhematological predisposing conditions. *J Clin Microbiol.* 53(2): 611–617.

Huang P, Jiang X, Wu B, Sun J. 2020. *Aspergillus jilinensis* sp. nov. and its thermostable alkaline enzymes evaluation. *Mycoscience.* 61(5): 205–211.

Hubka V, Nováková A, Kolařík M, Jurjević Ž, Peterson SW. 2015. Revision of *Aspergillus* section *Flavipedes*: Seven new species and proposal of section *Jani* sect. nov. *Mycologia.* 107(1): 169–208.

Samson RA, Peterson SW, Frisvad JC, Varga J. 2011. New species in *Aspergillus* section *Terrei*. *Stud Mycol.* 69(1): 39–55.

Samson RA, Visagie CM, Houbraken J, Hong SB, Hubka V, et al. 2014. Phylogeny, identification and nomenclature of the genus *Aspergillus*. *Stud Mycol.* 78(1): 141–173.

Shen S, Liu SL, Jiang JH, Zhou LW. 2021. Addressing widespread misidentifications of traditional medicinal mushrooms in *Sanghuangporus* (Basidiomycota) through ITS barcoding and designation of reference sequences. *IMA Fungus.* 12(1).

Visagie CM, Houbraken J. 2020. Updating the taxonomy of *Aspergillus* in South Africa. *Stud Mycol.* 95(1): 253–292.

Wu F, Zhou LW, Vlasák J, Dai YC. 2022. Global diversity and systematics of *Hymenochaetaceae* with poroid hymenophore. *Fungal Divers.* 113(1): 1–192.
