# Supplementary material for: FunVIP: Fungal Validation and Identification Pipeline based on phylogenetic analysis
Source: J Microbiol. 2025 Apr 29;63(4):e2411017. doi: 10.71150/jm.2411017 (PMC13577050; doi:10.71150/jm.2411017)
Supplement: Supplementary file 20 [file jm-2411017-Supplementary-Data-7.docx]

**Supple Data 7. Mean and standard deviation values for accuracy, precision, recall, and F1 score of FunVIP, BLAST, and q2-feature-classifier using the *Sanghuangporus* and *Aspergillus* section *Terrei* datasets**

The Mann–Whitney U test was used to compare the results of each pair of analyses (Supple Table 7.1**–**4).

**Table S7.1.** Mean and standard deviation values for accuracy, precision, recall, and F1 score for FunVIP, BLAST, and q2-feature-classifier by *Sanghuangporus* dataset

|  | **Mean** | | | | **Standard deviation** | | | |
| --- | --- | --- | --- | --- | --- | --- | --- | --- |
|  | **Accuracy** | **Precision** | **Recall** | **F1** | **Accuracy** | **Precision** | **Recall** | **F1** |
| **FunVIP fast** | 0.9742 | 0.8243 | 0.8053 | 0.8089 | 0.0042 | 0.0434 | 0.0313 | 0.0335 |
| **FunVIP accurate** | 0.9694 | 0.8436 | 0.7916 | 0.8003 | 0.0212 | 0.0443 | 0.0439 | 0.0429 |
| **BLAST ITS** | 0.8635 | 0.7988 | 0.7463 | 0.7352 | 0.1611 | 0.0438 | 0.1202 | 0.1056 |
| **q2-feature-classifer ITS** | 0.9567 | 0.8403 | 0.7904 | 0.8048 | 0.0203 | 0.0254 | 0.0170 | 0.0143 |

**Table S7.2.** Mean and standard deviation values for accuracy, precision, recall, and F1 score for FunVIP, BLAST, and q2-feature-classifier by *Aspergillus* section *Terrei* dataset

|  | **Mean** | | | | **Standard deviation** | | | |
| --- | --- | --- | --- | --- | --- | --- | --- | --- |
|  | **Accuracy** | **Precision** | **Recall** | **F1** | **Accuracy** | **Precision** | **Recall** | **F1** |
| **FunVIP fast** | 0.9859 | 0.8929 | 0.8994 | 0.8959 | 0.0040 | 0.0079 | 0.0017 | 0.0053 |
| **FunVIP fast *BenA*** | 0.9972 | 0.9844 | 0.9883 | 0.9858 | 0.0080 | 0.0440 | 0.0331 | 0.0401 |
| **FunVIP fast *CaM*** | 0.8776 | 0.6713 | 0.6905 | 0.6781 | 0.0189 | 0.0663 | 0.0804 | 0.0720 |
| **FunVIP accurate** | 0.9944 | 0.9806 | 0.9851 | 0.9816 | 0.0121 | 0.0468 | 0.0373 | 0.0448 |
| **FunVIP accurate *BenA*** | 0.9972 | 0.9844 | 0.9883 | 0.9858 | 0.0080 | 0.0440 | 0.0331 | 0.0401 |
| **FunVIP accurate *CaM*** | 0.8987 | 0.7362 | 0.7805 | 0.7518 | 0.0000 | 0.0000 | 0.0000 | 0.0000 |
| **BLAST *BenA*** | 0.9494 | 0.8262 | 0.8844 | 0.8310 | 0.0215 | 0.0026 | 0.0280 | 0.0164 |
| **BLAST *CaM*** | 0.8847 | 0.6695 | 0.7275 | 0.6821 | 0.0193 | 0.0813 | 0.0473 | 0.0741 |
| **q2-feature-classifier *BenA*** | 0.9634 | 0.7620 | 0.7798 | 0.7687 | 0.0111 | 0.0332 | 0.0378 | 0.0337 |
| **q2-feature-classifier *CaM*** | 0.8903 | 0.5993 | 0.6254 | 0.6082 | 0.0119 | 0.0311 | 0.0405 | 0.0331 |

**Table S7.3.** Results of the Mann-Whitney U test result for each pair of analyses in the *Sanghuangporus* dataset

|  | **BLAST ITS** | **FunVIP accurate** | **FunVIP fast** | **q2-classifier ITS** |
| --- | --- | --- | --- | --- |
| **BLAST ITS** | 1.0E+00 | 1.9E-03 | 3.3E-04 | 2.3E-02 |
| **FunVIP accurate** | 1.9E-03 | 1.0E+00 | 1.3E-01 | 2.1E-02 |
| **FunVIP fast** | 3.3E-04 | 1.3E-01 | 1.0E+00 | 4.2E-02 |
| **q2-classifier ITS** | 2.3E-02 | 2.1E-02 | 4.2E-02 | 1.0E+00 |

**Table S7.4.** Results of the Mann-Whitney U test result for each pair of analyses in the *Aspergillus* section *Terrei* dataset

|  | **BLAST *BenA*** | **BLAST *CaM*** | **FunVIP accurate** | **FunVIP accurate *BenA*** | **FunVIP accurate *CaM*** | **FunVIP fast** | **FunVIP fast *BenA*** | **FunVIP fast *CaM*** | **q2-classifier *BenA*** | **q2-classifier *CaM*** |
| --- | --- | --- | --- | --- | --- | --- | --- | --- | --- | --- |
| **BLAST *BenA*** | 1.0E+00 | 7.5E-04 | 5.1E-04 | 1.6E-04 | 4.3E-04 | 1.6E-04 | 1.6E-04 | 4.8E-04 | 1.8E-01 | 5.7E-04 |
| **BLAST *CaM*** | 7.5E-04 | 1.0E+00 | 2.3E-04 | 1.8E-04 | 2.1E-01 | 1.8E-04 | 1.8E-04 | 2.2E-01 | 3.1E-04 | 5.0E-01 |
| **FunVIP accurate** | 5.1E-04 | 2.3E-04 | 1.0E+00 | 5.9E-01 | 9.4E-05 | 8.4E-03 | 5.9E-01 | 2.2E-04 | 1.3E-03 | 2.3E-04 |
| **FunVIP accurate *BenA*** | 1.6E-04 | 1.8E-04 | 5.9E-01 | 1.0E+00 | 7.0E-05 | 2.2E-03 | 1.0E+00 | 1.7E-04 | 3.7E-04 | 1.8E-04 |
| **FunVIP accurate *CaM*** | 4.3E-04 | 2.1E-01 | 9.4E-05 | 7.0E-05 | 1.0E+00 | 7.0E-05 | 7.0E-05 | 1.2E-02 | 1.4E-04 | 3.3E-02 |
| **FunVIP fast** | 1.6E-04 | 1.8E-04 | 8.4E-03 | 2.2E-03 | 7.0E-05 | 1.0E+00 | 2.2E-03 | 1.7E-04 | 3.7E-04 | 1.8E-04 |
| **FunVIP fast *BenA*** | 1.6E-04 | 1.8E-04 | 5.9E-01 | 1.0E+00 | 7.0E-05 | 2.2E-03 | 1.0E+00 | 1.7E-04 | 3.7E-04 | 1.8E-04 |
| **FunVIP fast *CaM*** | 4.8E-04 | 2.2E-01 | 2.2E-04 | 1.7E-04 | 1.2E-02 | 1.7E-04 | 1.7E-04 | 1.0E+00 | 3.0E-04 | 2.7E-01 |
| **q2-classifier *BenA*** | 1.8E-01 | 3.1E-04 | 1.3E-03 | 3.7E-04 | 1.4E-04 | 3.7E-04 | 3.7E-04 | 3.0E-04 | 1.0E+00 | 3.1E-04 |
| **q2-classifier *CaM*** | 5.7E-04 | 5.0E-01 | 2.3E-04 | 1.8E-04 | 3.3E-02 | 1.8E-04 | 1.8E-04 | 2.7E-01 | 3.1E-04 | 1.0E+00 |
