# Supplementary material for: FunVIP: Fungal Validation and Identification Pipeline based on phylogenetic analysis
Source: J Microbiol. 2025 Apr 29;63(4):e2411017. doi: 10.71150/jm.2411017 (PMC13577050; doi:10.71150/jm.2411017)
Supplement: Supplementary file 21 [file jm-2411017-Supplementary-Data-8.docx]

**Supple Data 8.** **Example of the polytomy-resolving process performed using FunVIP**

To assess the effectiveness of the polytomy-resolving process, we compared FunVIP results obtained with and without polytomy-resolving process. To generate FunVIP result without polytomy-resolving, the FunVIP result directory of the *Aspergillus* section *Terrei* dataset, using the accurate preset and distance cutoff 0.01, was copied and was rerun (See “500_Polytomy_resolve” directory of the archived data in Zenodo repository). Original trees and edited ITS trees from both runs were compared to evaluate performance of the polytomy-resolving process. The polytomy-resolving process successfully rearranged zero-length branches and resulted in a clearer final visualization (Fig. S8.1).

**Fig. S8.1.** Example of the polytomy-resolving process performed with FunVIP. ITS trees from the *Aspergillus* section *Terrei* dataset are compared by applying the polytomy-resolving option.
